# Facile saccharide-free mimetics that recapitulate key features of glycosaminoglycan sulfation patterns†
†Electronic supplementary information (ESI) available. See DOI: 10.1039/c8sc02303d


**DOI:** 10.1039/c8sc02303d

**Published:** 2018-08-24

**Authors:** Teck Chuan Lim, Shuting Cai, Roland G. Huber, Peter J. Bond, Priscilla Xian Siew Chia, Siv Ly Khou, Shujun Gao, Su Seong Lee, Song-Gil Lee

**Affiliations:** a Institute of Bioengineering and Nanotechnology , 31 Biopolis Way, The Nanos , Singapore 138669 , Singapore . Email: sglee@ibn.a-star.edu.sg; b Bioinformatics Institute , 30 Biopolis Street, #07-01 Matrix , Singapore 138671 , Singapore; c Department of Biological Sciences , National University of Singapore , 14 Science Drive 4 , Singapore 117543 , Singapore

## Abstract

Controlling glycosaminoglycan (GAG) activity to exploit its immense potential in biology ultimately requires facile manipulation of sulfation patterns associated with GAGs. However, satisfying this requirement in full remains challenging, given that synthesis of GAGs is technically arduous while convenient GAG mimetics often produce sulfation patterns that are uncharacteristic of GAGs. To overcome this, we develop saccharide-free polyproline-based GAG mimetics (PGMs) that can be facilely assembled *via* amide coupling chemistry. Molecular dynamics simulations show that PGMs recapitulate key GAG structural features (*i.e.* ∼9 Å-sized repeating units, periodicity and helicity) and as with GAGs, can be tuned to introduce systematic variations in sulfate clustering and spacing. Functionally, a variety of PGMs control various GAG activities (concerning P-selectin, neurotrophic factors and heparinase) and exhibit GAG-like characteristics such as progressive modulation, comparable effectiveness with heparins, need for different sequences to suit different activities and the presence of a “minimal bioactive length”. Furthermore, PGMs produce consistent effects *in* vivo and successfully provide therapeutic benefits over cancer metastasis. Taken together with their high level of biosafety, PGMs answer the long-standing need for an effective and practicable strategy to manipulate GAG-appropriate sulfation patterns and exploit GAG activity in medicine and biotechnology.

## Abstract 2

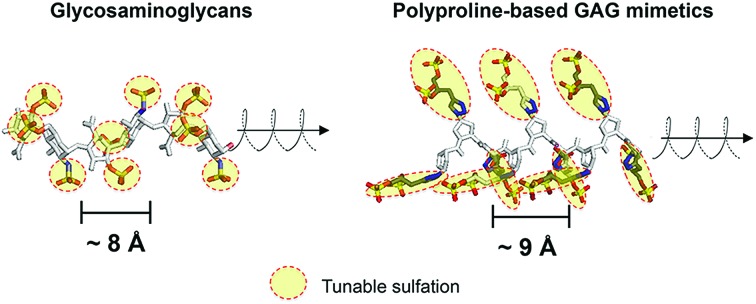
We report a new class of saccharide-free glycosaminoglycan (GAG) mimetics where polyproline imparts facilely-made sulfation patterns with GAG-like structure, function and tunability.

## Introduction

**Fig. 1:**
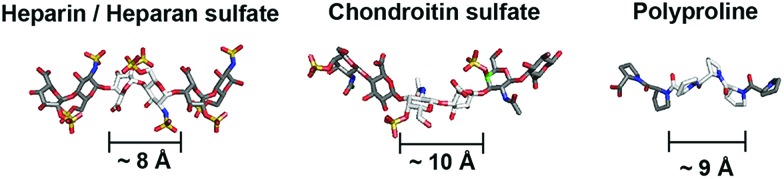
Schematic to illustrate similarities in the molecular architecture of natural GAGs and polyprolines (heparin from PDB: 1HPN, chondroitin sulfate from PDB: ; 4N8W, polyproline from CCDC: 1014542).

Glycosaminoglycans (GAGs) encode variable sulfation patterns by utilizing different sequences of repeating 8–10 Å-sized disaccharides to display sulfates moieties along and around their helical axis^1^ (Fig. 1). These sulfation patterns provide the primary basis for modulating interactions with proteins and mediating vital biological activities spanning across development,^2^ regeneration^3^ and cancer.^4^ One of the “holy grails” of glycobiology is the facile manipulation of sulfation patterns associated with GAGs to tune and exploit GAG activities in desired ways. Given the profound difficulty in extracting and purifying naturally occurring GAGs amidst their heterogeneity, the two main strategies to this end are the bottom-up synthesis of tailored GAG sequences and the creation of GAG mimetics. While valuable in their own right, these strategies often present a conundrum. Synthesized GAG sequences inherently encode defined sulfate-bearing motifs and sulfation patterns with appropriate characteristics but the synthesis and adjoining of GAG saccharides require sophisticated synthetic^5^,^6^ and/or chemo-enzymatic^7^ methods that are technically challenging and inaccessible to many. On the other hand, GAG mimetics, which predominantly focus on the use of non-carbohydrate backbones (*e.g.* polymers,^8^ dendrimers^9^,^10^ and linker-coupled polyphenols^11^), show the converse. They offer convenient means to generate sulfate-bearing motifs or sulfation patterns but they often depart significantly from the molecular architecture and characteristics of GAGs and fail to tap into the tunable framework that GAGs use to establish versatile control over their biological activity. Previously, we attempted to bridge these differences by employing selected elements of both strategies. Specifically, we created GAG mimetics with CS-E disaccharides as bioactive sulfate-bearing motifs and polyprolines as non-carbohydrate backbones that aimed to recover the characteristic of spatial tunability.^12^ Despite successes in producing GAG mimetics that could potentiate NGF/TrkA-induced neurogenesis, the challenge remains apparent. Arduous saccharide synthesis, while reduced, is still needed to a certain degree to create GAG-appropriate sulfate-bearing motifs. Sulfation patterns also remain uncharacteristic of GAGs. Like many GAG mimetics explored thus far, sulfate-bearing motifs are positioned unnaturally as pendants around the molecular backbone instead of following the natural framework of lining up continuously within the backbone.

Toward a resolution to the above, we develop a new strategy to achieve both GAG-appropriate sulfation patterns and chemical accessibility by emphasizing design of saccharide-free GAG mimetics to recapitulate key structural features of GAGs. To do so, we utilize polyproline, not merely to take advantage of its well-defined structure as before but also to exploit the unique characteristics of its polyproline II (PPII) conformation. In particular, three consecutive proline residues form a 9 Å-sized helical turn^13^ (Fig. 1). Conjugating sulfate moieties directly onto such residues thus presents an opportunity to create a sulfate-bearing motif that model GAG disaccharides in terms of length scale and radial projection of sulfate moieties. Such proline-based motifs can, moreover, be repeated end-to-end (rather than assembled as pendants) to organize sulfation patterns that adhere to a GAG-like helical, periodic format. Importantly, assembly of polyprolines involves readily accessible amide coupling chemistry. Proline residues, with or without sulfate moieties, can be easily and precisely concatenated in any desired order, thus raising the possibility to emulate GAGs in the ability to systematically encode variable sulfate-bearing motifs and overall sulfation patterns. In this report, we demonstrate that this new class of saccharide-free polyproline-based GAG mimetics (PGMs) provides facile means to generate sulfation patterns that closely resemble those of GAGs. Systematic variations in their sulfation patterns successfully control an assortment of GAG activities and produce consistent effects across *in vitro* and *in vivo* contexts concerning cancer metastasis. In addition, PGMs exhibit a remarkable safety profile. Overall, PGMs open up compellingly practicable avenues to tap the extensive potential of GAG activities for medical and biotechnological applications.

## Results

### Synthesis of saccharide-free PGMs

**Fig. 2:**
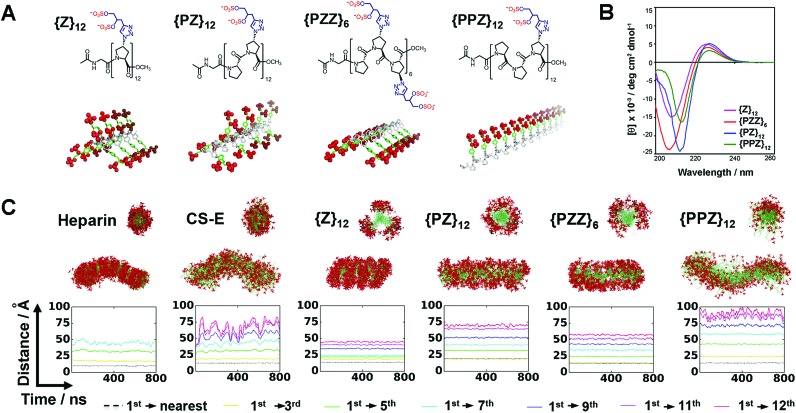
(A) Chemical structures and 3D depictions of PGMs. (B) CD spectra of PGMs. (C) MD simulation results showing the sulfation patterns for heparin, CS-E, **{Z}_12_**, **{PZ}_12_**, **{PZZ}_6_** and **{PPZ}_12_**. Top and bottom structures show respectively the axial cross-sectional view and overall view of superimposed frames over 800 ns. Plot shows the profile of inter-sulfate distances between 1^st^ and selected sulfated proline residues. Sulfate moieties in (A and C) are highlighted in red.

Direct coupling of sulfated proline monomers either suffers poor yield or requires cumbersome protection–deprotection steps. To synthesize PGMs, we conceived a 2-stage synthetic strategy that involved: (1) assembly of PGM precursors from proline, P, and azido-proline monomers, Z_A_; and (2) generation of the final PGMs by converting Z_A_ to sulfated proline, Z, *via* click reaction with alkyne-containing sulfate moieties (ESI Fig. S1 and S2†). As a proof of concept, we synthesized several distinct PGMs, namely **{Z}_12_**, **{PZ}_12_**, **{PZZ}_6_** and **{PPZ}_12_**, by varying the sequence of proline and sulfated proline residues (Fig. 2A). All PGMs contained exactly 24 sulfate moieties. For each PGM, HPLC and mass spectrometry confirmed precise assembly of the desired precursor (ESI Fig. S3–S6†). FT-IR and NMR analysis further verified complete modifications of the precursor and the integrity of sulfate moieties on the final PGMs (ESI Fig. S7–S11†). The chemical characterizations collectively show that PGMs avoid the issues of sequence heterogeneity and incomplete modifications found in naturally occurring GAGs. Dihydroxyl variants for these PGMs were also synthesized as unsulfated controls (ESI Fig. S1C, S7, S12–S15†). Finally, circular dichroism (CD) was performed for PGMs, their dihydroxyl variants, CS-E, heparin and tinzaparin (Fig. 2B, ESI Fig. S16†). For all PGMs and their dihydroxyl variants, CD spectra exhibited the 208–213 nm minima and 225–228 nm maxima characteristic of PPII conformation and ascertained the presence of the crucial PPII helical conformation regardless of the arrangement of sulfate moieties.

### PGMs produce GAG-like sulfation patterns

We employed molecular dynamics (MD) simulations to analyze and compare the sulfation patterns generated by natural GAGs and PGMs (Fig. 2C, Movies S1–S6†). The sulfation patterns among natural GAGs, namely heparin octasaccharide and chondroitin sulfate-E (CS-E) dodecasaccharide (both of which match the number of sulfate moieties in PGMs), were substantially different. For heparin, which is the most highly sulfated GAG and is known to be a relatively stiff helix,^14^ superposition of frames over 800 ns showed that the distribution of sulfate moieties was dense and had a band-like appearance. On the other hand for CS-E, sulfate moieties were relatively sparser and distributed more evenly around the molecular backbone. For both heparin and CS-E, inter-sulfate distances began around 10 Å and increased in a stepwise manner, thus reflecting the periodicity in their sulfation patterns.

For **{Z}_12_**, **{PZ}_12_**, **{PZZ}_6_** and **{PPZ}_12_**, MD simulations revealed a collection of sulfation patterns that relate closely to those borne by heparin and CS-E. In terms of the distribution of sulfate moieties, the PGMs displayed systematic variations that fell in between the dense and sparse distribution found in heparin and CS-E respectively. Inter-sulfate distances began around 10 Å for **{Z}_12_**, **{PZZ}_6_** and **{PPZ}_12_** and began correctly around 20 Å for **{PZ}_12_** (which had been designed for increased inter-sulfate spacing). All inter-sulfate distances increased in a stepwise manner, indicating a periodicity that resembles natural GAGs. A noted feature of natural GAGs is the variable circumferential clustering of sulfate moieties toward one or multiple sides of their helical backbone.^5^,^14^,^15^ Radial cross-sections of superimposed frames from MD simulations demonstrated that **{PPZ}_12_**, **{PZZ}_6_** and **{Z}_12_**/**{PZ}_12_** successfully changed the clustering of sulfate moieties to 1, 2 and 3 sides respectively. All in all, PGMs make possible the ability to produce and manipulate GAG-like sulfation patterns.

### PGMs modulate CS-E binding and tumor cell adhesion to P-selectin

**Fig. 3:**
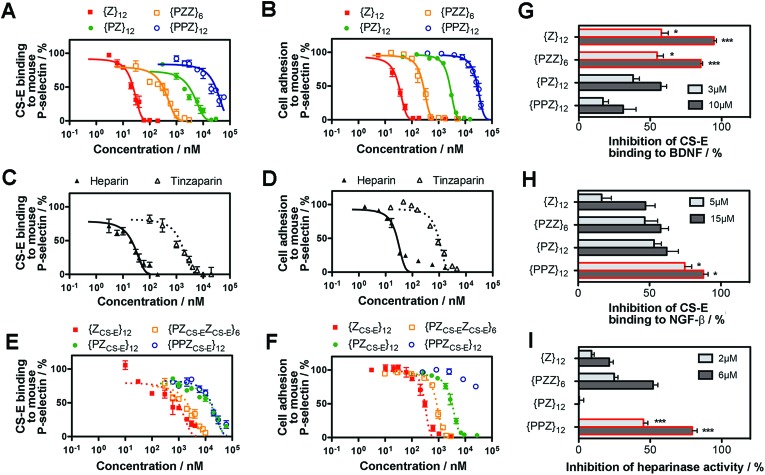
Ability of PGMs to modulate GAG activity. Inhibition of CS-E binding to mouse P-selectin by (A) PGMs (*n* = 4), (C) heparin and tinzaparin (*n* = 4) and (E) saccharide-based variants of PGMs (*n* = 4). Inhibition of adhesion of B16-F10 murine melanoma cells to mouse P-selectin by (B) PGMs (*n* = 9), (D) heparin and tinzaparin (*n* = 9) and (F) saccharide-based variants of PGMs (*n* = 9). (G) Inhibition of CS-E binding to human BDNF by PGMs (*n* = 6). (H) Inhibition of CS-E binding to human NGF-β by PGMs (*n* = 7). (I) Inhibition of heparinase activity by PGMs (*n* = 7). Data represent means ± SEM. Statistical analysis was performed by 1- or 2-way ANOVA with *post hoc* Bonferroni comparison test (*P < 0.05, ***P < 0.001).

We determined whether the sulfation patterns of PGMs were effective in controlling GAG activities. One of the predominant GAG activities that many seek to manipulate is the interactions of GAGs with cell adhesion molecules (CAMs). Interactions between GAGs (*e.g.* CS-E^16^) and P-selectin are of particular interest because they trigger and shape prominent pathophysiological events such as metastasis, thrombosis and inflammation.^17^–^19^ Given this, we tested the utility of PGMs in modulating CS-E binding to P-selectin. *In vitro* protein binding assays showed that switching across **{PPZ}_12_**, **{PZ}_12_**, **{PZZ}_6_** and **{Z}_12_** progressively inhibited CS-E binding to mouse and human P-selectin (Fig. 3A, ESI Fig. S17A, ESI Tables S1 and S2†). Interaction of cell surface chondroitin sulfate with P-selectin can mediate adhesion of tumor cells (ESI Fig. S18†). To ascertain the utility of PGMs in the context of cells, we further used them to inhibit the adhesion of B16-F10 and A375 melanoma cells to mouse and human P-selectin respectively. Again, switching PGMs in the same order of **{PPZ}_12_**, **{PZ}_12_**, **{PZZ}_6_** and **{Z}_12_** increased the ability to inhibit tumor cell adhesion to immobilized P-selectin (Fig. 3B, ESI Fig. S17B, ESI Tables S3 and S4†). In all these assays, **{Z_U_}_12_**, which is a unsulfated, dihydroxyl variant of **{Z}_12_**, did not show any inhibitory effects and demonstrated the critical role of sulfate moieties (ESI Fig. S19†).

To gain insights into how the different PGMs might produce the trend observed above, we simulated unbiased ensembles to observe how CS-E or PGMs would interact with human P-selectin (ESI Fig. S20A†). From random initial positions, both CS-E and PGMs gravitated toward and engaged a large, shallow surface on P-selectin that was enriched with basic residues (ESI Fig. S20B†). Per-residue analyses on MM-GBSA interaction energies and closeness of contact (ESI Fig. S20C and D†) indicated that the interactions of CS-E with P-selectin focused on four clusters of basic residues, namely {Lys8, Arg16, Lys17, Arg22}, {Arg54, Lys55, Lys58, Lys66, Lys67}, {Lys84, Arg85} and {Lys111, Lys112, Lys113}, and exhibited emphasis on the first two clusters. **{Z}_12_** and **{PZZ}_6_** followed these interactions closely, but **{Z}_12_** was better than **{PZZ}_6_** in their emphasis on the first two clusters. On the other hand, **{PZ}_12_** showed diminished interactions for the latter two clusters and **{PPZ}_12_** showed diminished interactions for all four clusters. These analyses suggest that through their variable structures and spatial organization of sulfate moieties, PGMs modulated the manner of engaging basic residues on P-selectin and in doing so, inhibited CS-E binding differently.

We further asked how the sulfation patterns of PGMs fared without having actual saccharides. First, we compared the effectiveness of PGMs to that of commonly used natural GAGs such as heparin (∼77–86 sulfate moieties) and tinzaparin (clinical-grade, low-molecular-weight heparin; ∼25–34 sulfate moieties). Comparison of IC_50_ for inhibiting CS-E binding showed that the most effective PGM, **{Z}_12_**, was comparable to heparin for mouse P-selectin and outperformed tinzaparin for both human and mouse P-selectin (Fig. 3C and D, ESI Fig. S21A and B, ESI Tables S1–S4†). Second, we compared the PGMs with our previous mimetics that contained actual CS-E disaccharides as pendant groups (ESI Fig. S22–S28†). Strikingly, the saccharide-free PGMs were overall more effective and established clearer functional differences with variations in their sulfation patterns than the saccharide-based mimetics (Fig. 3E and F, ESI Fig. S29A and B, ESI Tables S1–S4†). Third, we explored whether PGMs could reproduce a hallmark of GAG sequences *i.e.* the existence of a “minimal bioactive length” at and above which the activity increases drastically.^20^,^21^ For this, we synthesized length variants to **{Z}_12_** (ESI Fig. S30–S38†) and compared across all of them. Our results showed that IC_50_ dropped drastically by over 1000-fold from **{Z}_6_** to **{Z}_12_** and decreased marginally by ∼10-fold with further elongation to **{Z}_18_** and **{Z}_24_**, indicating the existence of a “minimal bioactive length” at **{Z}_12_** (ESI Fig. S39, ESI Tables S5 and S6†). All these showed that the saccharide-free sulfation patterns generated by the PGMs were viable with regard to P-selectin.

### Applicability of PGMs toward neurotrophic factors and heparinase

Next, we assessed the utility of PGMs beyond CAMs by employing them to modulate CS-E binding to neurotrophic factors such as brain-derived-neurotrophic factor (BDNF) (Fig. 3G) and nerve growth factor-beta (NGF-β) (Fig. 3H) and to regulate the enzymatic activity of heparinase (Fig. 3I). In the case of NGF-β for which we had previously tested saccharide-based mimetics, the required concentrations of PGMs appeared higher than what might be expected, suggesting that the absence of saccharides may lead to lower affinity. Nonetheless, in all the cases examined, systematic variations in the sulfation patterns of PGMs successfully controlled GAG activity. Again, sulfate moieties are essential components of PGMs because unsulfated variants (*i.e.***{Z_U_}_12_**, **{PZ_U_Z_U_}_6_**, **{PZ_U_}_12_** and **{PPZ_U_}_12_**) failed to produce any observable effects (ESI Fig. S40†). Our data also revealed that the control over GAG activity was typically progressive rather than all-or-nothing. For example, 10 μM of **{PPZ}_12_**, **{PZ}_12_**, **{PZZ}_6_** and **{Z}_12_** inhibited CS-E binding to BDNF by 31%, 57%, 85% and 95% respectively. This is in line with the widely recognized disposition of GAGs to provide fine-tuning rather than to drive binary outcomes.^1^ Moreover, the trend across the various PGMs varied with the biological event. For the inhibition of CS-E binding, the trend for BDNF was **{PPZ}_12_** < **{PZ}_12_** < **{PZZ}_6_** ≈ **{Z}_12_** but reversed almost completely in the case of NGF-β to **{Z}_12_** < **{PZZ}_6_** < **{PZ}_12_** < **{PPZ}_12_**. Yet for inhibition of heparinase activity, the order became **{PZ}_12_** < **{Z}_12_** < **{PZZ}_6_** < **{PPZ}_12_**. Finally, when we explored using PGMs to inhibit heparin binding to NGF-β, the trend changed again to **{PPZ}_12_** ≈ **{PZ}_12_** ≈ **{PZZ}_6_** < **{Z}_12_** (ESI Fig. S41†). All these demonstrate that similar to natural GAGs, the sulfation patterns of PGMs can take into account the changing requirements across different events.

### Translational potential of PGMs

**Fig. 4:**
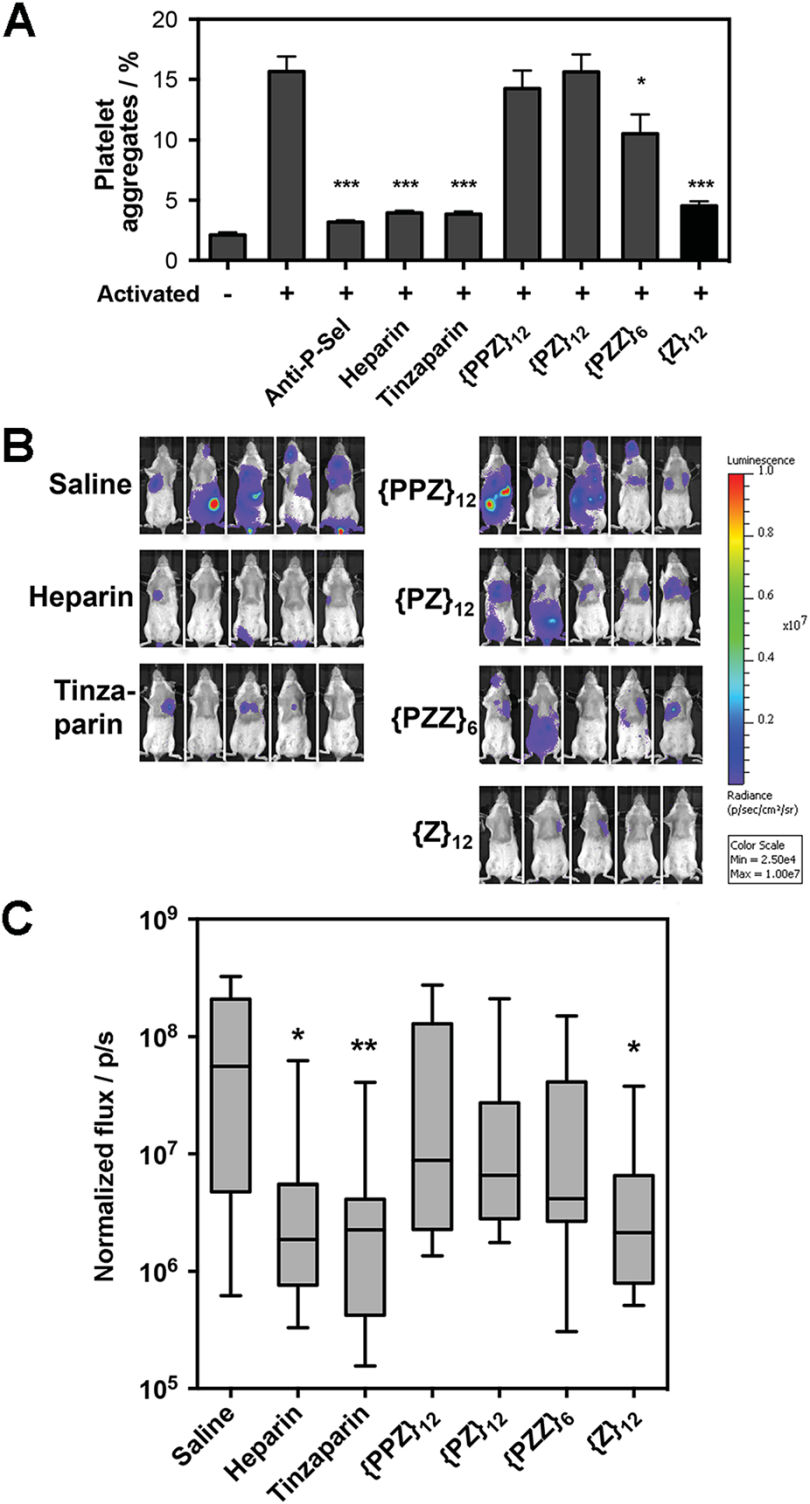
Translational potential of PGMs. (A) Aggregation of activated mouse platelets in the presence of saline, anti-mouse P-selectin, heparin, tinzaparin and PGMs (*n* = 9). Data represent means ± SEM. (B) Representative whole mouse bioluminescence images of B16-F10 Red-Fluc metastasis and (C) quantification of tumor burden upon treatment with heparin, tinzaparin and PGMs (*n* = 13). Box plot shows median with min to max. Statistical analysis was performed by 1-way ANOVA with *post hoc* Bonferroni comparison test in (A) and by Kruskal–Wallis analysis with Dunn's comparison test in (C) (*P < 0.05, **P < 0.01, ***P < 0.001).

Along our lines of investigation with P-selectin, we employed PGMs to modulate the physiological event of platelet aggregation, which was linked to increased surface expression of P-selectin (ESI Fig. S42†) and could be reduced by anti-P-selectin antibody (Fig. 4A, ESI Fig. S43†). Consistent with the *in vitro* findings above, flow cytometry results showed that switching across **{PPZ}_12_**, **{PZ}_12_**, **{PZZ}_6_** and **{Z}_12_** increasingly reduced platelet aggregation and could eventually reach similar effectiveness as heparin and tinzaparin. To study relevance to pathological events, we further employed PGMs *in vivo* to inhibit hematogenous metastasis. A single dose of heparin administered shortly before the introduction of tumor cells into the bloodstream has already been found to attenuate hematogenous metastasis in mice primarily by inhibiting P-selectin.^22^ Upon verifying that PGMs themselves did not affect viability of tumor cells (ESI Fig. S44†), we used a similar murine model and regimen and performed bioluminescence imaging to quantify tumor burden at 2 weeks after the intravenous injection of luciferase-expressing B16-F10 Red-Fluc cells. Our data revealed that manipulation of sulfation patterns across **{PPZ}_12_**, **{PZ}_12_**, **{PZZ}_6_** and **{Z}_12_** repeated the uptrend *in vivo* to the point where a single dose of **{Z}_12_** could reduce metastasis as effectively as heparin and tinzaparin (Fig. 4B and C). Overall, the translational potential of PGMs is evident in terms of: (1) yielding a lead candidate whose sulfation pattern can impact physiological and pathological events with sufficient therapeutic benefit; and more importantly, (2) how the different sulfation patterns of PGMs produce consistent effects across *in vitro* and *in vivo* contexts.

### Biosafety of PGMs

**Fig. 5:**
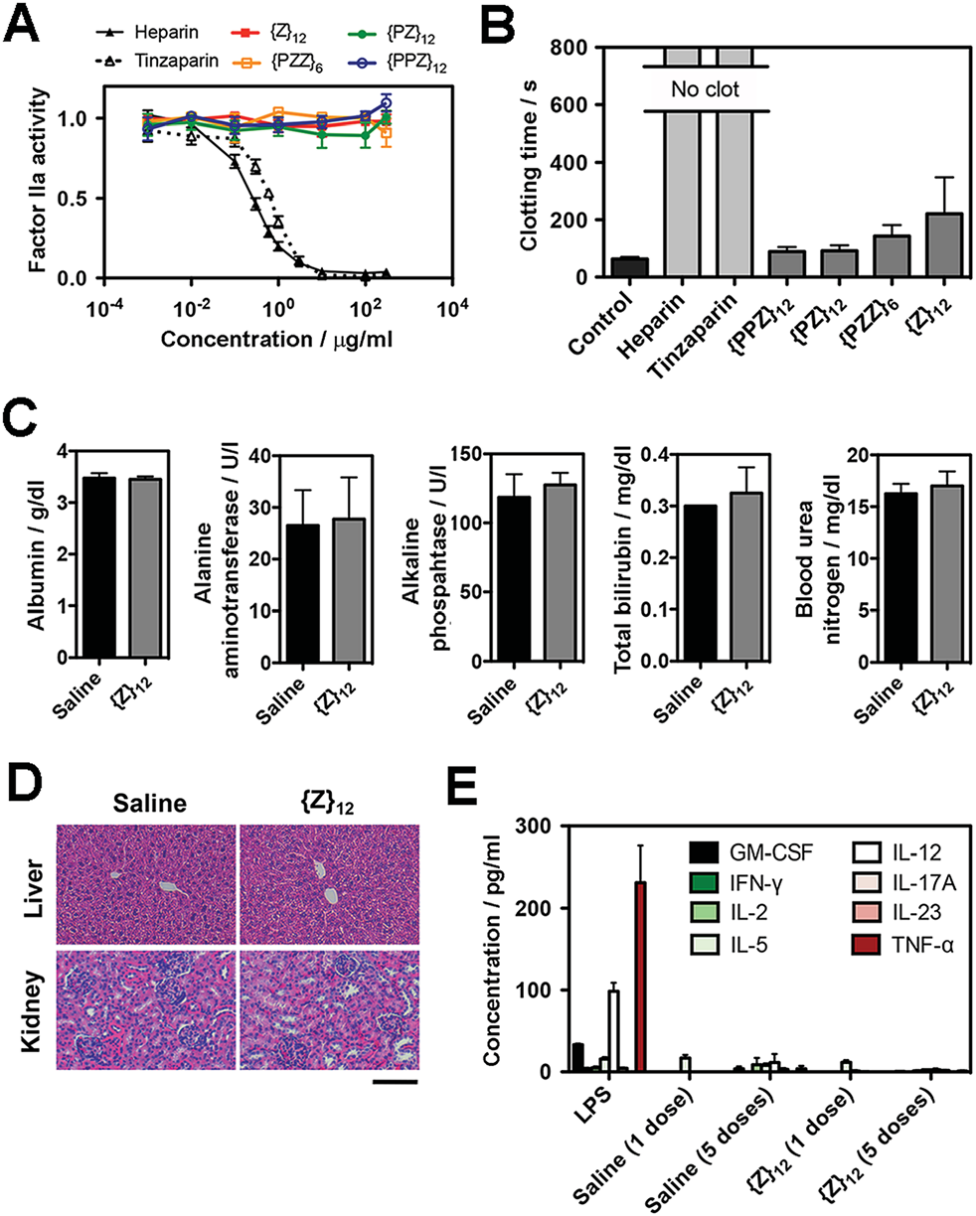
(A) Factor IIa activity (*n* = 4) and (B) clotting time of whole mouse blood (*n* = 5 for heparins, *n* = 6 for all others) to evaluate anticoagulation effects of heparin, tinzaparin and PGMs. (C) Levels of albumin, alanine aminotransferase, alkaline phosphatase, bilirubin and urea nitrogen in blood 7 days after intravenous injection of saline or **{Z}_12_** (*n* = 4 mice). (D) Representative H&E stain of liver and kidney at 1 week to demonstrate *in vivo* safety of **{Z}_12_** (*n* = 4 mice). (E) Levels of pro-inflammatory cytokines in mouse blood serum at 2 h after single dose of LPS, saline and **{Z}_12_** and after the last of 5 doses of saline and **{Z}_12_** spaced over a month (*n* = 5 mice). Scale bar: 100 μm. Data for (A–C), (E) represent means ± SEM.

Finally, we assessed the biosafety of PGMs, which is requisite to taking advantage of all the merits demonstrated above. A leading concern is anti-coagulant activity as a potential off-target side effect, since heparin, heparan sulfate and even sulfated small molecules^23^ can activate antithrombin III which in turn inhibit proteases (*e.g.* factor IIa and Xa) in the blood coagulation cascade. Enzyme activity assays showed that all PGMs did not share the inhibitory activity of heparin and tinzaparin on factor IIa and Xa (Fig. 5A, ESI Fig. S45†). Further testing with whole mouse blood showed that coagulation was completely inhibited with heparin and tinzaparin but proceeded reasonably well with PGMs (Fig. 5B). We also checked the lead candidate, **{Z}_12_**, for adverse effects upon intravenous injection into mice. **{Z}_12_** did not induce whole body weight loss (ESI Fig. S46†), elevate markers of liver damage (Fig. 5C) or cause histopathological abnormalities in liver and kidney (Fig. 5D). Moreover, while immunogenic molecules such as lipopolysaccharides (LPS) strongly elevated pro-inflammatory cytokines in the bloodstream, **{Z}_12_** barely induced any cytokine elevation whether it was used as a single dose or administered repeatedly for 5 times over a longer term of one month (Fig. 5E). All these indicate that PGMs can be safely employed.

## Discussion

To date, attempts on defined means to control GAG activity typically straddle between two options. One option is chemical^5^,^6^ or chemo-enzymatic^7^ synthesis of specific GAG sequences. This is the most direct way to access and manipulate the sulfation patterns that underpin GAG activities but it requires iterations of intricate control over stereoselectivity and regioselectivity.^24^ Even for the creation of short GAG sequences for the fabrication of GAG mimetics, this can be difficult to be employed on an extensive scale. The other option is to use non-saccharide GAG mimetics. Currently, such mimetics focus on the convenience of non-saccharide molecular backbones^8^–^11^ to carry sulfate moieties more than their structural resemblance to GAGs. However, the role of the sulfation patterns that underpin the myriad of vital GAG activities is not merely to house an adequate number of sulfate moieties but more importantly, to organize sulfate moieties spatially along the molecular architecture imparted by the saccharide-based backbone of GAGs. Reports have shown the importance of recognizing this molecular architecture in terms of the defined periodic spacing between sulfate moieties^25^ and the helical layout of sulfate moieties.^26^ In numerous studies, manipulating specific O- and N-sulfates within GAGs modulate their interactions with proteins,^6^,^27^ highlighting that sulfate moieties positioned appropriately within the confines of the saccharide-based layout are key to the control of GAG activity. Analysis of GAG-binding domains among proteins and peptides has also revealed consensus spacing^28^,^29^ and topological motifs^30^ among the basic residues, again pointing to the issue of certain order and structure within sulfation patterns of GAGs.

Polyproline can be facilely assembled with easily accessible amide coupling chemistry. When we developed PGMs to exploit polyproline directly for the organization of sulfate moieties, we discovered that PGMs recapitulate key (although not all) structural features of GAGs (*i.e.* periodicity, helicity, length scale of the repeating units and tunability) and allowed for controlled variations to the circumferential clustering and axial spacing of the sulfate moieties. This mean a new option of relying on the facile manipulation of PGM sequences to produce a systematic variety of GAG-like sulfation patterns.

Our data revealed that for GAG activities associated with certain proteins (*e.g.* P-selectin), the saccharide-free sulfation patterns of PGMs were similar, if not better, than saccharide-based ones. For other proteins (*e.g.* NGF-β), saccharides may not be fully dispensable for binding affinity and their absence needs to be compensated with high doses of PGMs. Nevertheless, in all cases, the sulfation patterns (or in other words, the 3D presentation of sulfate moieties) of PGMs were consistently a crucial factor in controlling GAG activity. We found the control of GAG activity by PGMs to be notable in the several ways. First, all the PGMs studied here span over ∼50–100 Å, which is approximately the length of 8 to 20-mer GAG sequences. Unlike GAG mimetics that present sulfate moieties on small molecules^31^ or rely on long-range arrangements of sulfate moieties over sizeable polymers and dendrimers,^10^,^32^ PGMs arrange sulfate moieties over length scales that are typical of basic residue clusters on protein surfaces. Such a way of controlling GAG activity can associate closely with the workings of natural GAGs. Second, PGMs demonstrate a certain extent of selectivity. Different PGMs stand out for different proteins-of-interest (*e.g.* P-selectin *vs.* heparinase) or for inhibiting different types of GAGs (*e.g.* CS-E *vs.* heparin). Third, all PGMs carry the same number of sulfate moieties and have the versatility to control GAG activity solely through differences in their sulfation patterns. We emphasize that this departs from many attempts (*e.g.* modifications of GAGs^33^ and generation of differently sized sulfated polymers^10^) where controlling and dissecting the true value of sulfation patterns from the intertwined changes in the number of sulfate moieties is difficult. Fourth, PGMs generate sulfation patterns in a continuous fashion along their backbone axis. This relates better to GAGs than our previous attempt^12^ and the typical GAG mimetic paradigm of introducing short GAG sequences as pendant groups around a non-saccharide backbone.^34^,^35^

Much interesting work lies ahead in our efforts to further develop this PGM-based paradigm. While we explored and investigated a small number of PGM sequences as a proof-of-concept in this study, any sequence is possible. Generating a significant diversity of sequences will be useful to reveal and exploit the full extent of selectivity that PGMs can offer. The current PGMs also use a binary format where each proline is either undecorated or carrying a pair of sulfated moieties. Conceivably, PGMs can be advanced into higher-order formats through altering the number of sulfate moieties per proline residue and exploring different architectures of the linker between proline and sulfated moieties. Non-ionic moieties (*e.g.* polar and hydrophobic groups) can also be included to take advantage of various secondary interactions found between natural GAGs and proteins.^36^,^37^

## Conclusion

We have developed saccharide-free polyproline-based GAG mimetics (PGMs) that make possible the ability to achieve both GAG-appropriate sulfation patterns and chemical accessibility. PGMs display GAG-like structure, function and versatility. They modulate various GAG activities (concerning P-selectin, neurotrophic factors and heparinase), produce consistent effects *in vivo* to offer therapeutic benefits and exhibit a high degree of biosafety. We expect PGMs to offer an effective and practical paradigm for developing therapeutics and materials that require appropriately tuned GAG activity or a systematic spectrum of GAG activities to produce beneficial outcomes.

## Experimental details

ESI Fig. S1–S46, ESI Tables S1–S7, Movies S1–S6,† detailed description of all experimental procedures and characterization of all new compounds are given in the in the ESI.†

## Live subject statement

All animal experiments were approved by the Institutional Animal Care and Use Committee (Agency for Science, Technology and Research, Singapore) and performed in strict accordance with the Institutional guidelines. All procedures were performed in accordance with the Institutional Animal Care and Use Committee (Agency for Science, Technology and Research, Singapore).

## Conflicts of interest

There are no conflicts to declare.

## Supplementary Material

Supplementary information

Supplementary movie

Supplementary movie

Supplementary movie

Supplementary movie

Supplementary movie

Supplementary movie
