# Supplementary material for: Facile saccharide-free mimetics that recapitulate key features of glycosaminoglycan sulfation patterns
Source: Chem Sci. 2018 Aug 24;9(41):7940–7. doi: 10.1039/c8sc02303d (PMC6201788; doi:10.1039/c8sc02303d)
Supplement: Supplementary file 1 [file SC-009-C8SC02303D-s001.pdf]

Electronic Supplementary Material (ESI) for Chemical Science.
This journal is © The Royal Society of Chemistry 2018

Facile Saccharide-free Mimetics that Recapitulate the Key Features of Glycosaminoglycan Sulfation Patterns

Teck Chuan Lim, Shuting Cai, Roland G. Huber, Peter J. Bond, Priscilla Xian Siew Chia, Siv Ly Khou, Shujun Gao, Su Seong Lee, and Song-Gil Lee*

Electronic supplementary information

(Materials and methods, Fig. S1-S46, Tables S1-S7, Captions for Movies S1–S6, Supplementary References)

General methods

Unless otherwise stated, reactions were performed using anhydrous solvents and in flame-dried glassware under argon atmosphere. All commercial reagents were used as received unless otherwise stated. Thin layer chromatography (TLC) was performed using E. Merck silica gel 60 F254 precoated plates (0.25 mm) and visualization of the developed chromatogram was performed by UV, cerium ammonium molybdate, or ninhydrin stain as necessary. Merck silica gel 60 (particle size 0.040 - 0.063 mm) was used for flash chromatography. Peptide purification was conducted on Gilson HPLC GX-271 System equipped with a reverse phase Kromasil® 100-5-C18 column (21.2 x 250 mm). Gel filtration chromatography (Sephadex G-15 or LH-20 ultrafine: GE Healthcare) was used for the purification of polyproline-based glycosaminoglycan mimetics (PGMs).

^1H NMR spectra were recorded on a Bruker AVIII 400 (400 MHz) spectrometer and are reported in parts per million (δ) relative to D_2O (4.79 ppm). Data for the ^1H NMR spectra are reported as follows: chemical shift (δ ppm), multiplicity (s = singlet, bs = broad singlet, t = triplet, m = multiplet), coupling constant in Hz, and integration. ^{13}C NMR spectra were obtained on a Bruker AVIII 400 (100 MHz) spectrometer and are reported in terms of chemical shift. Mass spectra were obtained from Waters SQD Quadrupole Mass Spectrometer equipped with Waters Acquity™ Ultra Performance Liquid Chromatography (column: Waters Acquity UPLC® BEH C18 1.7 µm (1.0 x 150 mm)) and were recorded in m/z. HPLC conditions were: 1) 5% A over 0.5 min; 2) 5-60% A over 5.5 min; 3) 60-80% A over 0.5 min and; 4) 80% A over 1.5 min (where A: 99.9% CH_3CN / 0.1%TFA, B: 90% H_2O / 9.9% CH_3CN / 0.1% TFA, %A + %B = 100%, flow rate = 0.3 mL/min). High resolution ESI mass spectra were obtained from Chemical,

Molecular and Materials Analysis Centre at the National University of Singapore. FT-IR spectra were collected using Perkin-Elmer Spectrum 100 and are reported in terms of wavenumber (cm^{-1}).

Abbreviation

SO_3•TMA	Sulfur trioxide trimethylamine complex
Ac_2O	Acetic anhydride
DIPEA	N,N-Diisopropylethylamine
TBTU	N,N,N',N'-Tetramethyl-O-(benzotriazol-1-yl)uranium tetrafluoroborate
HOBt	Hydroxybenzotriazole
TFA	Trifluoroacetic acid
TBTA	Tris[1-benzyl-1H-1,2,3-triazol-4-yl)methyl]amine

Synthesis of pent-4-yne-1,2-diyl bis(sulfate) (2).

To a solution of pent-4-yne-1,2-diol **1** (100 mg, 1.0 mmol) in DMF (5.8 mL) was added SO_3•TMA (804 mg, 6.0 mmol). The mixture was stirred at 50 °C overnight under argon atmosphere. The product was purified by size exclusion chromatography (Sephadex LH-20 with MeOH/CH_2Cl_2 (1:1)), followed by silica gel chromatography (5% → 50% MeOH:CH_2Cl_2). Pent-4-yne-1,2-diyl bis(sulfate) **2** was obtained as a white solid (257 mg, 99%, protonated form) (Figure S2). HRMS (ESI) m/z: $[M - H]^-$ calcd for $C_5H_6O_9S_2$, 258.9582; found, 258.9587.

General procedure for the synthesis of PGM precursors.

All PGM precursors were prepared *via* standard Boc chemistry in the solution phase as reported previously.[1] Briefly, to a solution of Boc-Peptide(1)-OH (1.0 equiv.) in DMF were added H-Peptide(2)-OCH_3 (1.1 equiv.), TBTU (1.1 equiv.), and DIPEA (5.0 equiv.) (final concentration: 0.1M). The mixture was stirred at room temperature until the reaction was completed. After the completion, the solvent was removed *in vacuo* to afford a yellow solid. Purification of this solid by either flash column chromatography on silica or reverse phase HPLC afforded Boc-Peptide(1)-Peptide(2)-OCH_3. To deprotect methyl ester group, aq. NaOH (2.0 equiv.) was added to a solution of Boc-Peptide(1)-Peptide(2)-OCH_3 (1.0 equiv.) in THF/MeOH (1:1, final concentration was adjusted to 0.05M) and sonicated for 5 min. At this time, the same volume of CH_2Cl_2 was added and the reaction was stirred for additional 2 hrs. It was then neutralized with Amberlyst IR-120 resin, filtered, and evaporated *in vacuo* to afford Boc-Peptide(1)-Peptide(2)-OH quantitatively. N-termini of polypeptides were modified with acetyl group by treatment of Ac_2O (1.2 equiv.) and DIPEA (5.0 equiv.) in DMSO at room temperature for 1 hr. Purification of crude compounds by reverse phase HPLC afforded desired final PGM precursors as a white solid.

General procedure for the click reaction.

Pent-4-yne-1,2-diyl bis(sulfate) **2** (1.3 equiv. per azide), PGM precursor (1.0 equiv.), and TBTA (0.3 equiv. per azide) were added into a vial. Under argon atmosphere, the mixture was dissolved in anhydrous DMSO (final concentration: 0.1 M) and copper (I) iodide stock solution in DMSO (0.3 mol% per azide) and DIPEA (48.0 equiv.) were sequentially

added. The reaction mixture was then stirred for 14 days at room temperature under argon atmosphere. After complete consumption of Z_A units, the solvent was removed with continuous nitrogen flow. The resulting mixture was dissolved in 200 μL of 4M aq. NaCl and purified by Sephadex G-15 column (100% H_2O). Upon lyophilization, the desired PGMs were afforded as a white solid.

The azide vibrational band (~2100 cm^{-1}) in FTIR spectra was used to monitor the completion of the click reaction. FTIR was conducted using a Perkin Elmer FTIR Spectrum 100 between 4000 and 800 cm^{-1} at a spectral resolution of 4 cm^{-1}, with 4 scans per sample. Preparation of the FTIR was done by placing the samples on a germanium stage. The samples were then pressed before the measurement. The disappearance of the azide vibrational band in the spectra of the PGMs indicated the completion of the coupling reactions (Figure S7, S16, S27).

Circular dichroism (CD) analysis.

CD spectra were obtained using a Jasco-815 CD spectrometer equipped with a Peltier temperature controller (Jasco PTC-423S/15). Sample solutions of PGMs and tinzaparin were prepared at 200 μM while sample solutions of CS-E and heparin were prepared at 40 and 70 μM respectively. All sample solutions in 10 mM sodium phosphate-dibasic buffer (pH 7.0) were equilibrated at 4 °C for 24 hr, followed by at room temperature for 1 hr before measurements. Spectra were recorded at 25 °C from 260 to 200 nm. Mean residue ellipticity $[\theta]$ was calculated as follows;

$$[\theta] = \theta/(10 \cdot N \cdot c \cdot l)$$

θ represents the ellipticity in millidegrees, N the number of amino acid residues, c the

molar concentration in mol·L^{-1}, and *l* the cell path length in cm (Figure 2c; Figure S21, S31).

MD simulations.

Modified proline residues were constructed using PyMol.[2] Subsequent two-step quantum mechanical minimization at the theory levels PM6/aug-cc-pVDZ and wB97XD/aug-cc-pVDZ using Gaussian 09 Rev. C.01[3] yielded starting geometries. Subsequently, partial charges were derived using the AM1-BCC methodology [4] as implemented in Amber14[5] *tleap*. Charges were derived with an acetyl-cap on the N-terminal side and a N-methyl cap on the C-terminal side respectively. Subsequent removal of these caps yielded the final residue topology. Bonded terms were assigned according to the Amber force field ff14SB.[6] *ff14SB* parameters were chosen over GAFF[7] in order to seamlessly integrate the new residue with arbitrary default amino acids. Ligands were then constructed using the *sequence* command in *tleap*. In the case of the PGMs, the N-terminus consists of an acetyl-capped glycine residue followed by each of the four sequences of proline and modified proline residues and are capped with a methoxy-group on the C-terminus. Parameters for chondroitin sulfate-E (CS-E) were derived following the same protocol.

PGMs and CS-E were solvated as an extended conformation in cubic boxes with a wall separation of 0.6 nm using TIP3P water [8] and charges were neutralized using sodium counter ions parameterized according to Joung and Cheatham[9]. Electrostatics were described using the particle mesh Ewald procedure[10] with a long-range cutoff of 0.8 nm coinciding with truncation for Lennard-Jones interactions. The systems were optimized

for 2000 steps of steepest descent followed by 2000 steps of conjugate gradient minimization. Subsequently, the systems were equilibrated for 1 ns to constant density at 1 bar using a Berendsen barostat[11]. Langevin thermostatization [12] was used to achieve a constant temperature of 300 K. Bonds to hydrogen were constrained to allow a simulation time step of 2 fs. All calculations were performed using the GPU implementation of *pmemd.*[13] Molecular dynamics simulations of all systems were then performed for 800 ns and frames were saved at an interval of 100 ps. Comparison of proline ring chi angles with reference values from canonical up and down conformations[14] revealed all-down puckerings and therefore, showed consistency with PPII conformation[15] (Table S7).

In order to visualize the dynamics of the different PGMs and gauge their relative conformational flexibility, we aligned the ligands on their respective backbones. In the case of PGMs, this is the polyproline backbone. In the case of CS-E, the alignment was performed on the 6 heavy atoms within the pyranose substructure of the individual sugars. Subsequent to alignment, frames were extracted at 10 ns intervals and superimposed to visualize the conformational space explored by each PGM or CS-E. Additionally we calculated the distances of the center of mass of the charged sulfate groups with those of neighboring residues. The distance plots include measurements between sulfates on selected residues and the first sulfate-bearing proline residue.

Unbiased docking simulations.

The structure of human P-selectin was extracted from the PDB structure 1G1S.[16] In the absence of experimental structural information on either CS-E or PGM complexes with P-selectin, we conducted an extensive series of molecular dynamics simulations inspired by an dynamic molecular docking (DMD) approach reported previously for protein-GAG systems.[17] Unlike the protein-GAG systems described in the reported DMD approach, P-selectin does not have an apparent binding pocket or groove and the selection of an entry direction for the DMD pulling process may be biased. To avoid such bias, we instead created 100 independent starting geometries by placing human P-selectin at the center of a 7x7x7 nm cubic box and randomly inserting the respective ligands using the 'insert-molecules' tool of the Gromacs suite[18] (ESI Fig. S20A†). Next, we ran simulations of 10 ns each for all 100 starting conformations for each system using pmemd with implicit solvation,[19] using igb=5, mbondi2 radii[20] and a salt concentration of 0.15 M. We then conducted MM-GBSA calculations[21] on 100 frames of the final 1 ns of these simulations and focused our analysis on the top ten poses as ranked by MM-GBSA analyses (ESI Fig. S20B†). We calculated a closeness map for individual residues by assessing the inverse distance between the center of mass of each sulfate and the backbone atom of each protein residue (ESI Fig. S20C†). We also calculated per-residue energy decomposition from our MM-GBSA calculations to assess the energetic contributions of different regions of P-selectin (ESI Fig. S20D†).

Competitive ELISA.

CS-E (Cat. No. 400678) was purchased from AMS Biotechnology. Heparin (Cat. No. H3149) and Tinzaparin (Cat. No. T1490000) were purchased from Sigma-Aldrich. All

steps for competitive ELISA were performed at room temperature unless otherwise stated. To immobilize mouse and human P-selectin, 96-well high protein-binding plates (Nunc) were incubated overnight with 100 µl of 4 µg/ml Protein A (Thermo Fisher Scientific) at 4 °C, blocked with 1% bovine serum albumin (BSA) for 30 min and coated with 50 µl of 1 µg/ml recombinant mouse P-selectin-IgG1 or 2 µg/ml recombinant human P-selectin-IgG1 (R&D Systems) for 3 hr. To immobilize human BDNF and NGF-β, 96-well high protein-binding plates were directly coated with 50 µl of 4 µg/ml recombinant human BDNF or 50 µl of 10 µg/ml recombinant human NGF-β (Peprotech) for 3 hrs. Each well was then incubated for 1 hr with 100 µl solution where varying concentrations of heparin, tinzaparin or PGMs each competed against: 1) 5 nM biotin-CS-E for mouse P-selectin, human P-selectin as well as human BDNF; 2) 20 nM biotin-CS-E for human NGF-β and; 3) 30nM biotin-heparin for human NGF-β. For detection of biotin-CS-E or biotin-heparin bound to the immobilized proteins, each well was incubated with 100 µl Anti-Biotin-FITC (Miltenyi Biotec Cat. No. 130-090-857, dilution 1:40) for 1 hr and fluorescence measurements (Ex/Em: 490/525 nm) were measured using a microplate reader (Tecan Infinite M200 Pro). IC_{50} values were determined by non-linear regression.

Inhibition of tumor cell adhesion to immobilized P-selectin.

The assay was performed as described previously[22] using B16-F10 Red-Fluc murine melanoma cells (Perkin Elmer) and A375 human melanoma cells (ATCC). In Fig. S18, B16F10-luc-G5 cells were incubated for 1 hr with 0.5 U/ml chondroitinase ABC (Sigma Aldrich) beforehand. After the final wash, 10 µl cell lysis reagent (Promega) was added. Fluorescence measurements and IC_{50} were then obtained as per competitive ELISA.

Heparinase activity assay.

10 mU/ml heparinase I/III blend was used to digest 0.8 μg/ml tinzaparin at 37 °C for 22 hrs in the presence of 2 μM and 6 μM PGMs. 40 μl samples were analyzed using BIOPHEN Heparin Anti-IIa kit (HYPHEN BioMed; see method for *blood coagulation assay*) to evaluate the ability of heparinase to diminish tinzaparin-mediated attenuation of factor IIa activity.

Platelet aggregation assay.

Mouse whole blood was collected by cardiac puncture into acid-citrate-dextrose (66.6 mM citric acid monohydrate, 85 mM trisodium citrate dehydrate, 111 mM D-glucose) solution and centrifuged at 100 × g (10 min, room temperature) to produce platelet rich plasma (PRP). Apyrase (Sigma Aldrich) was added to PRP to a final concentration of 0.5 U/ml. PRP was incubated for 15 min and subsequently centrifuged at 800 × g (15 min, room temperature) on a 40% BSA cushion to obtain a platelet pellet. Platelets were resuspended in Tyrode's buffer (137 mM NaCl, 2.7 mM KCl, 12 mM $NaHCO_3$, 0.4 mM NaH_2PO_4, 5.5mM D-glucose, 10 mM HEPES, 0.35% BSA pH 7.3) to a final density of 10^5/μl. 100 μl of the platelet suspension was incubated for 5 min with (final concentration): (i) 5 μg/ml of PE or biotin-conjugated anti-mouse CD62P (Miltenyi Biotec 130-105-536 and 130-105-535 respectively), (ii) 200 nM PGMs, and (iii) 1.09 μg/ml heparin or tinzaparin (to match $\{\mathbf{Z}\}_{12}$ by mass concentration). Platelet aggregation was stimulated with 1 mM $MgCl_2$, 2 mM $CaCl_2$ and 6 nM phorbol 12-myristate 13-acetate at 37 °C for 15 min. Following this, platelet aggregates were fixed with 0.4%

paraformaldehyde for 5 min, diluted 4-fold in Tyrode's buffer and analyzed using a FACSAria II flow cytometer (BD Biosciences).

Cytotoxicity assay.

B16-F10 Red-Fluc murine melanoma cells were seeded (5000 per well) into a 96-well plate. After 24 hrs, the cells were incubated with serum-free RPMI for 1 hr. Subsequently, the medium was replaced with serum-free RPMI containing varying concentrations of heparin, tinzaparin and PGMs. Cell viability was evaluated at selected time points by measuring the metabolic activity of cells with PrestoBlue® Cell Viability Reagent (Thermo Fisher Scientific) according to manufacturer's instructions.

Experimental metastasis assay.

Solutions of heparin, tinzaparin and PGMs were prepared in Hank's Balanced Salt Solution (HBSS). B16-F10 Red-Fluc murine melanoma cells were cultured in RPMI/10% fetal bovine serum/1% penicillin-streptomycin, harvested with 1 mM EDTA and resuspended in HBSS at 2.5 × 10^5 cells per 100 µl. C57BL/6 albino mice (6 – 8 weeks old female) were randomized by sequential assignment to receive HBSS (vehicle control), heparin, tinzaparin or PGMs via tail vein injection. The doses were: 1) 150 µg, or equivalently 27.6 nmol, for $\{\mathbf{Z}\}_{12}$; 2) 150 µg for heparin and tinzaparin (to match $\{\mathbf{Z}\}_{12}$ by mass) and; 3) 27.6 mol for all other PGMs (to match $\{\mathbf{Z}\}_{12}$ by molar amount). Matching the dose of heparin and tinzaparin to $\{\mathbf{Z}\}_{12}$ by mass instead of concentration helped to ensure that the total number of sulfate moieties injected was similar with PGMs and that exact amounts of the polydisperse heparin and tinzaparin were used each time.

After 20 min, the mice were injected with 200 μl of the prepared tumor cell suspension. All injections were performed by an experimenter blinded to the treatment assigned to each mouse. Whole body weight of mice was measured on day 0 (before injection), 1, 3, 7 and 14. To evaluate metastasis on day 14, mice were anesthetized with isofluorane/O_2, administered Vivo-Glo™ luciferin (150 mg/kg in 200 μl PBS) via intraperitoneal injection and subjected to whole animal bioluminescence imaging using IVIS imaging system (Perkin Elmer).

Blood coagulation factor assay. BIOPHEN Heparin Anti-IIa and Anti-Xa (2 stages) kits (HYPHEN BioMed) were used according to manufacturer's instructions to measure the effect of heparin, tinzaparin or PGMs on activity of factor IIa and Xa. Briefly, 40 μl of the tested material at varying concentrations was sequentially mixed with: 1) anti-thrombin; 2) factor IIa or Xa and; 3) thrombin chromogenic substrate in a 96-well plate and incubated at 37 °C for 2 min after each mixing. The reaction was quenched with 80 μl of 20 g/L citric acid after 1 or 2 min for Anti-IIa and Anti-Xa assays respectively. The activity of factor IIa and Xa during the reaction was then evaluated via the measurement of absorbance at 405 nm.

Whole blood clotting analysis. Whole blood clotting time was measured using a MC10plus semi-automatic 10-channel ball coagulometer (ABW Medizin and Technik). Mouse whole blood was collected by cardiac puncture into 3.2% trisodium citrate. 100 μl of the citrated blood was mixed with 10 μl of PBS, heparin, tinzaparin or PGMs in a microtube. **{Z}$_{12}$** was tested at 75 μg/ml. Heparin and tinzaparin were tested at a matching

mass concentration while all other PGMs at a matching molar concentration. 100 μl of the mixture was transferred to a measuring cuvette and clotting was initiated with the addition of 100 μl 25 mM $CaCl_2$.

Assessment of *in vivo* toxicity. C57BL/6 albino mice (6 – 8 weeks old female) were administered HBSS (vehicle control) or 150 μg $\mathbf{\{Z\}_{12}}$ via tail vein injection. Mice that did not receive any injection were included as sham control. At week 1, blood was collected via retro-orbital bleeding into lithium-heparin tubes and analyzed using VetScan VS2 and comprehensive diagnostic profile rotors (Abaxis). Mice were further euthanized via CO_2 asphyxiation. Livers and kidneys were harvested, processed for histology and evaluated by a veterinary pathologist.

Measurement of blood serum cytokines. C57BL/6 albino mice (6 – 8 weeks old female) were administered HBSS (vehicle control) or 150 μg $\mathbf{\{Z\}_{12}}$ via tail vein injection. One group of mice received only a single dose while another group received 5 doses that were spaced over a month. Mice that received a single dose of 60 μg lipopolysaccharides (Sigma-Aldrich) were included as control. At 2 hr after the final dose, blood was collected via retro-orbital bleeding and left to clot at room temperature for 30 min. Serum was collected via centrifugation (10000 × g, 10 min, 4 °C) and assayed for cytokines using MACSPlex Cytokine 10 kit (Miltenyi Biotec) and MACSQuant Analyzer 10.

Statistical analysis. All replicates represent biological replicates. Unpaired *t*-tests or 1- or 2-way ANOVA with post-hoc Bonferroni comparison test was performed for the *in*

vitro experiments. Kruskal-Wallis analysis with Dunn's comparison test was performed for the animal study.

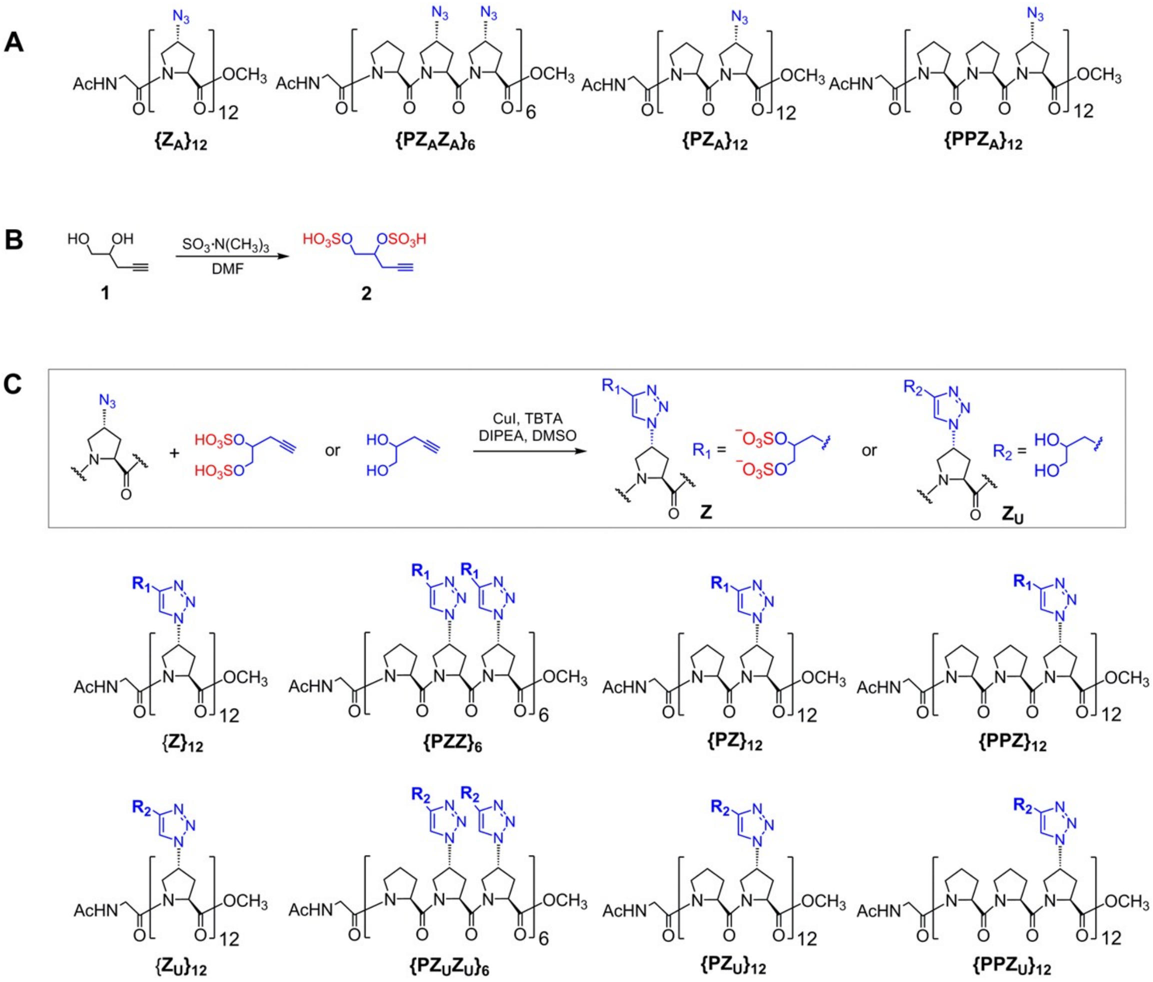

Fig. S1 (A) Structures of PGM precursors **{Z$_A$}$_{12}$**, **{PZ$_A$}$_{12}$**, **{PZ$_A$Z$_A$}$_6$** and **{PPZ$_A$}$_{12}$**, all of which bear a total of 12 {Z$_A$} units. (B) Synthesis of pent-4-yne-1,2-diyl bis(sulfate) **2**. (C) Click reaction for the synthesis of PGMs **{Z}$_{12}$**, **{PZ}$_{12}$**, **{PZZ}$_6$** and **{PPZ}$_{12}$** as well as the synthesis of their dihydroxyl variants **{Z$_U$}$_{12}$**, **{PZ$_U$}$_{12}$**, **{PZ$_U$Z$_U$}$_6$** and **{PPZ$_U$}$_{12}$**.

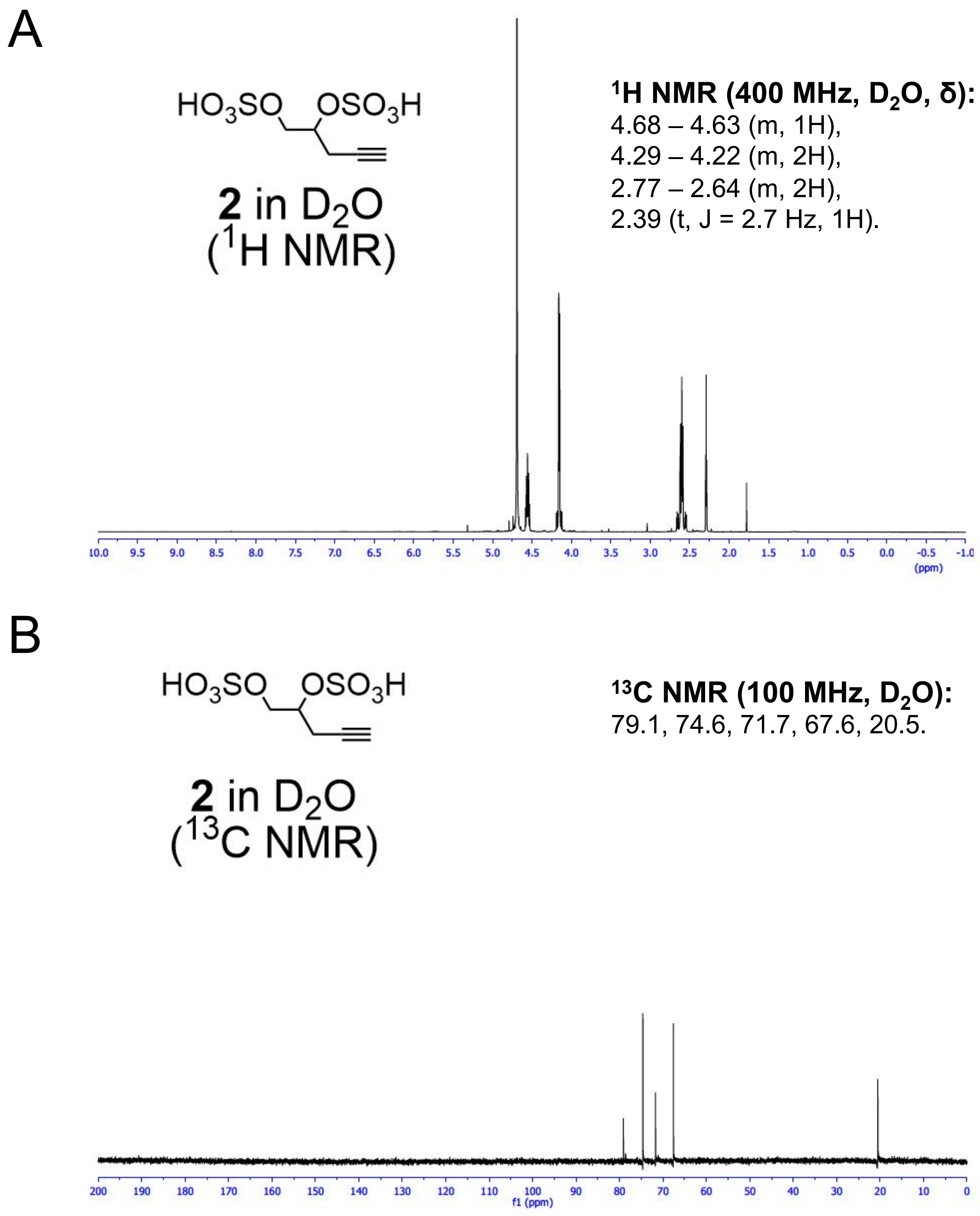

Fig. S2 (A) ^{1}H NMR spectra and (B) ^{13}C NMR spectra of pent-4-yne-1,2-diyl bis(sulfate) **2**.

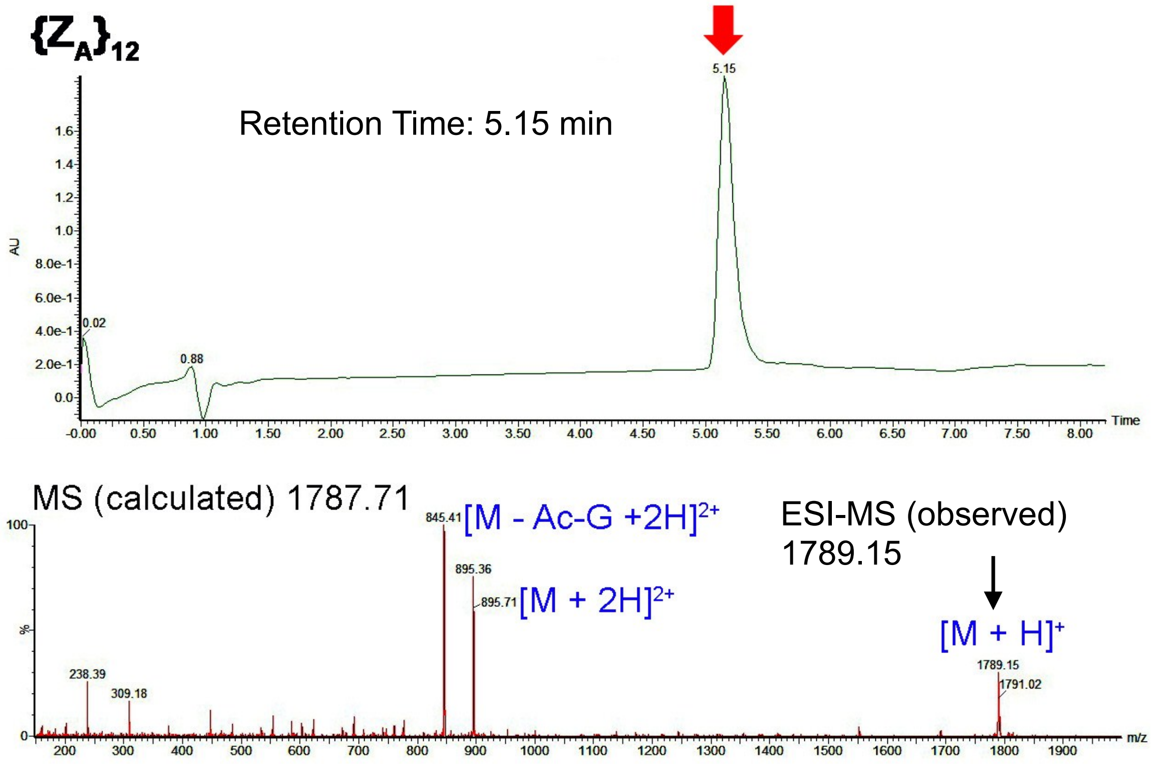

Fig. S3 Analytical HPLC traces (top) and ESI mass data (bottom) of **$\{Z_A\}_{12}$**.

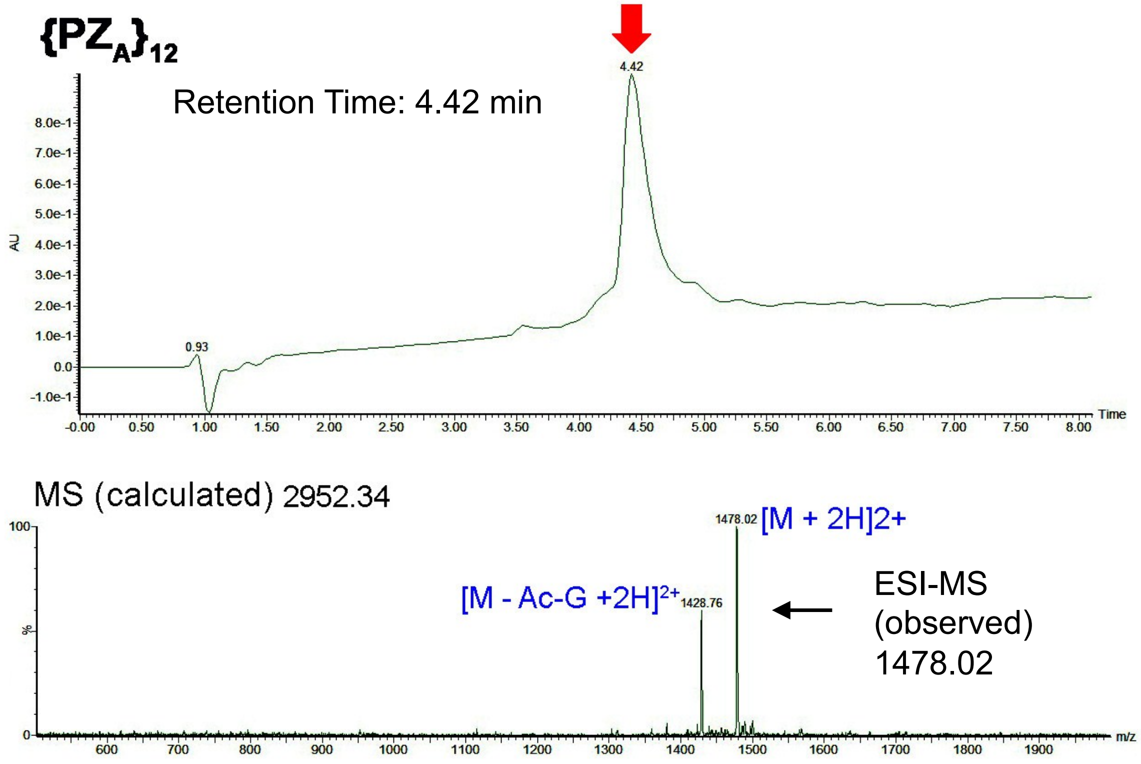

Fig. S4 Analytical HPLC traces (top) and ESI mass data (bottom) of **{PZ$_A$}$_{12}$**.

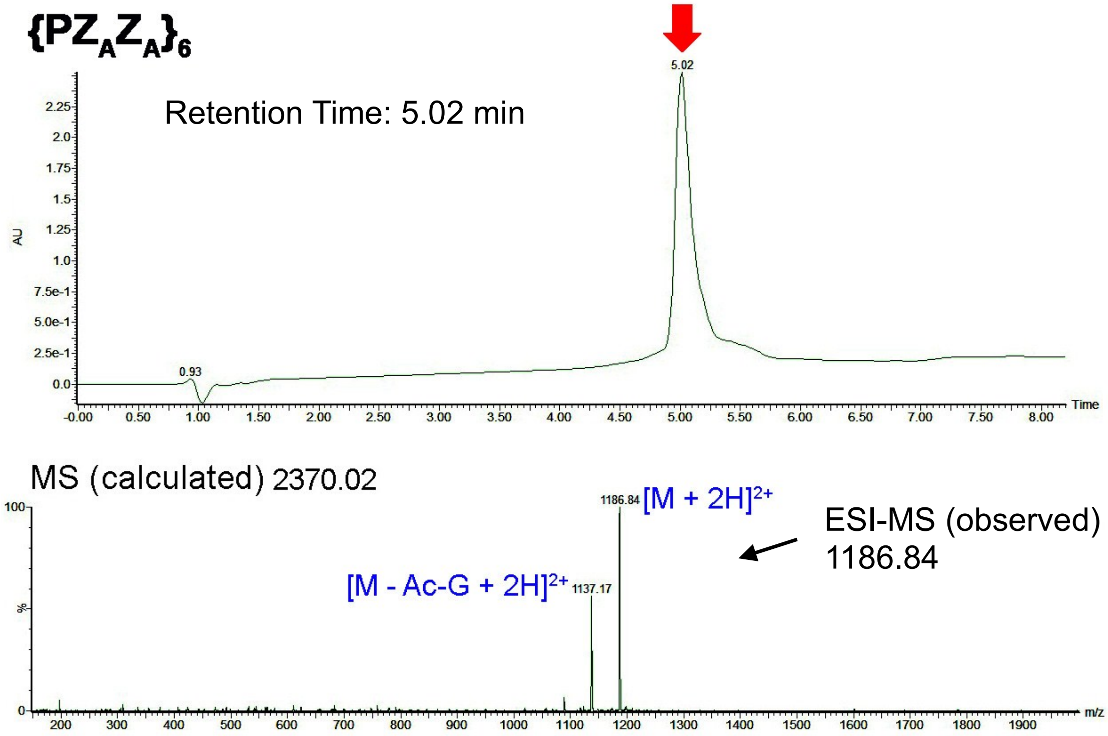

Fig. S5 Analytical HPLC traces (top) and ESI mass data (bottom) of $\{\mathbf{PZ_AZ_A}\}_6$.

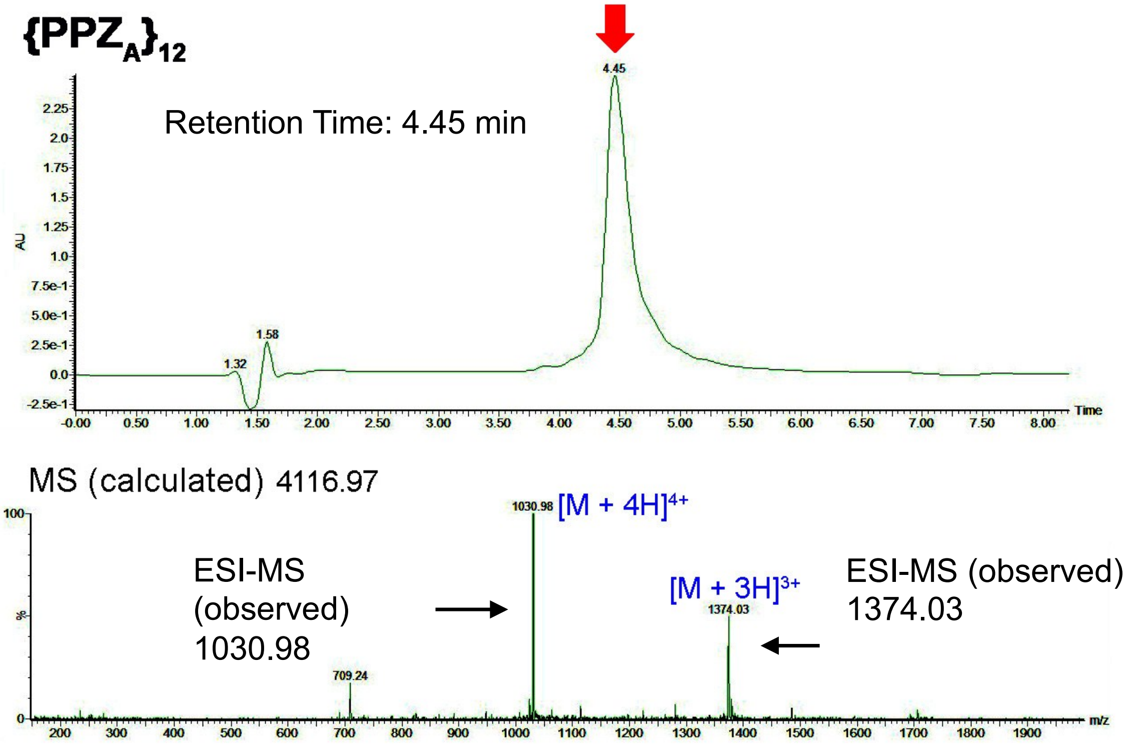

Fig. S6 Analytical HPLC traces (top) and ESI mass data (bottom) of **{PPZ$_A$}$_{12}$**.

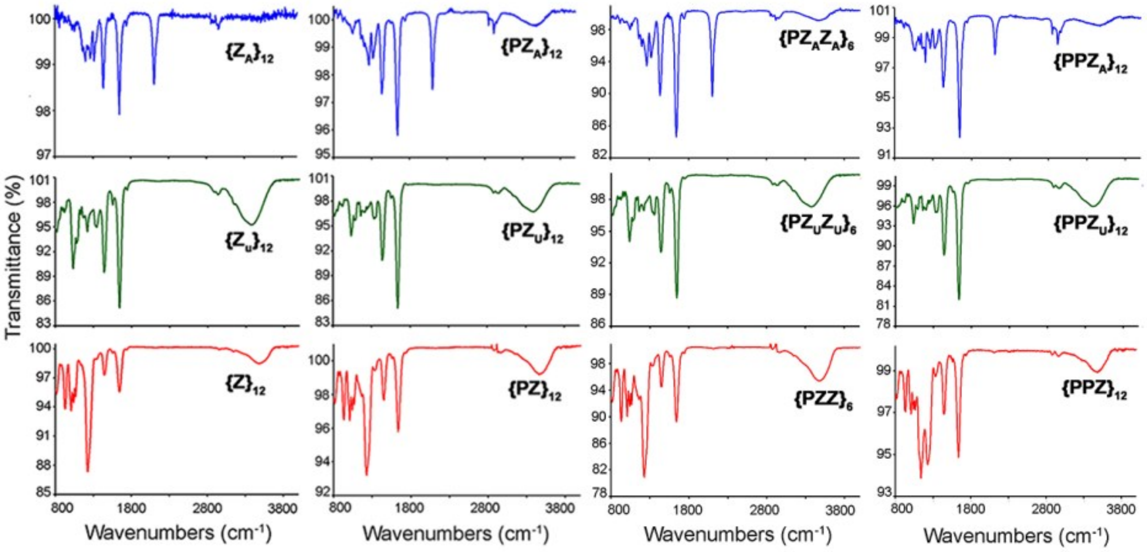

Fig. S7 FT-IR spectra of (blue) PGM precursors $\{\mathbf{Z_A}\}_{12}$, $\{\mathbf{PZ_A}\}_{12}$, $\{\mathbf{PZ_AZ_A}\}_6$ and $\{\mathbf{PPZ_A}\}_{12}$, (green) unsulfated, dihydroxyl variants of PGMs $\{\mathbf{Z_U}\}_{12}$, $\{\mathbf{PZ_U}\}_{12}$, $\{\mathbf{PZ_UZ_U}\}_6$ and $\{\mathbf{PPZ_U}\}_{12}$ and (red) sulfated PGMs $\{\mathbf{Z}\}_{12}$, $\{\mathbf{PZ}\}_{12}$, $\{\mathbf{PZZ}\}_6$ and $\{\mathbf{PPZ}\}_{12}$.

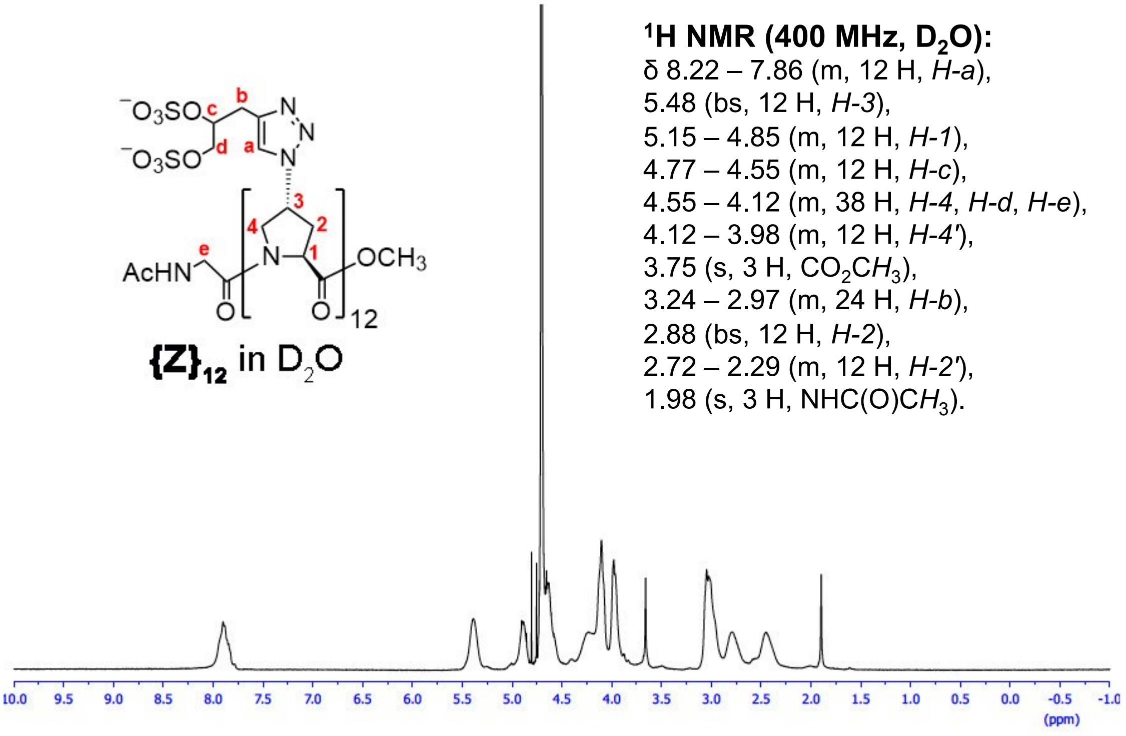

Fig. S8 ^{1}H NMR spectra of **$\{Z\}_{12}$**.

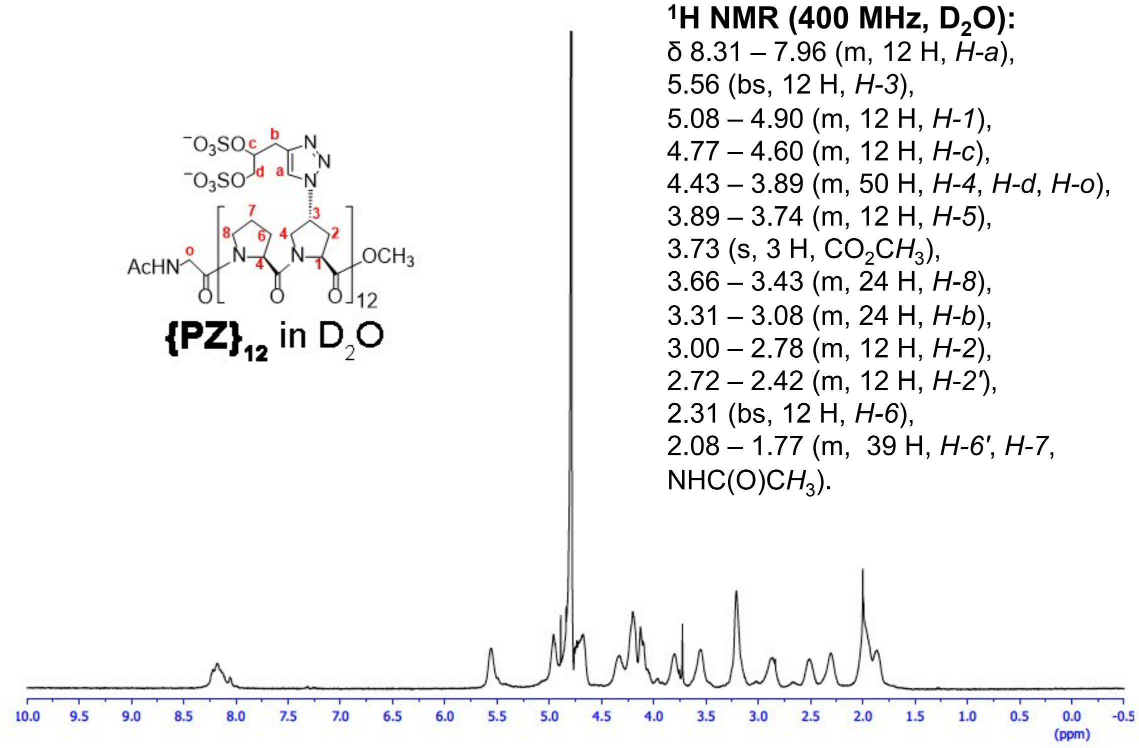

Fig. S9 ^{1}H NMR spectra of **{PZ}$_{12}$**.

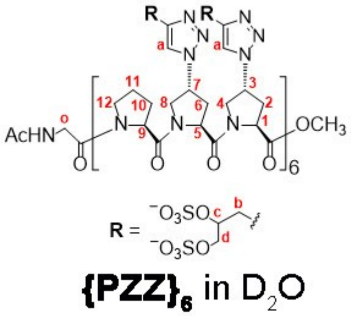

^{1}H NMR (400 MHz, D_2O):
δ 8.06 – 7.87 (m, 12 H, *H-a*),
5.47 (bs, 12 H, *H-3*, *H-7*),
5.03 – 4.90 (m, 12 H, *H-1*, *H-5*),
4.76 – 4.61 (m, 12 H, *H-c*),
4.41 – 3.94 (m, 50 H, *H-4*, *H-8*, *H-d*, *H-o*),
3.94 – 3.78 (m, 6 H, *H-9*),
3.75 (s, 3 H, CO_2CH_3),
3.68 – 3.45 (m, 12 H, *H-12*),
3.22 – 3.03 (m, 24 H, *H-b*),
2.96 – 2.75 (m, 12 H, *H-2*, *H-6*),
2.69 – 2.18 (m, 12 H, *H-2'*, *H-6'*),
2.11 – 1.81 (m, 27 H, *H-10*, *H-11*,
NHC(O)CH_3).

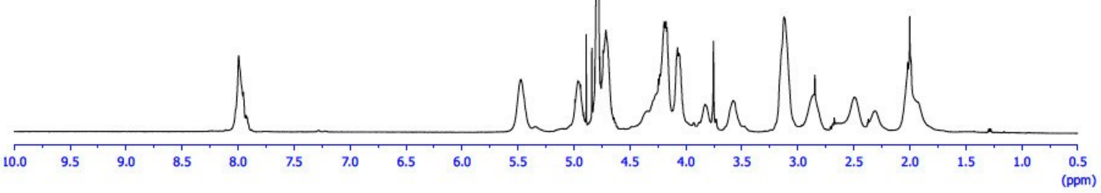

Fig. S10 ^{1}H NMR spectra of **{PZZ}$_6$**.

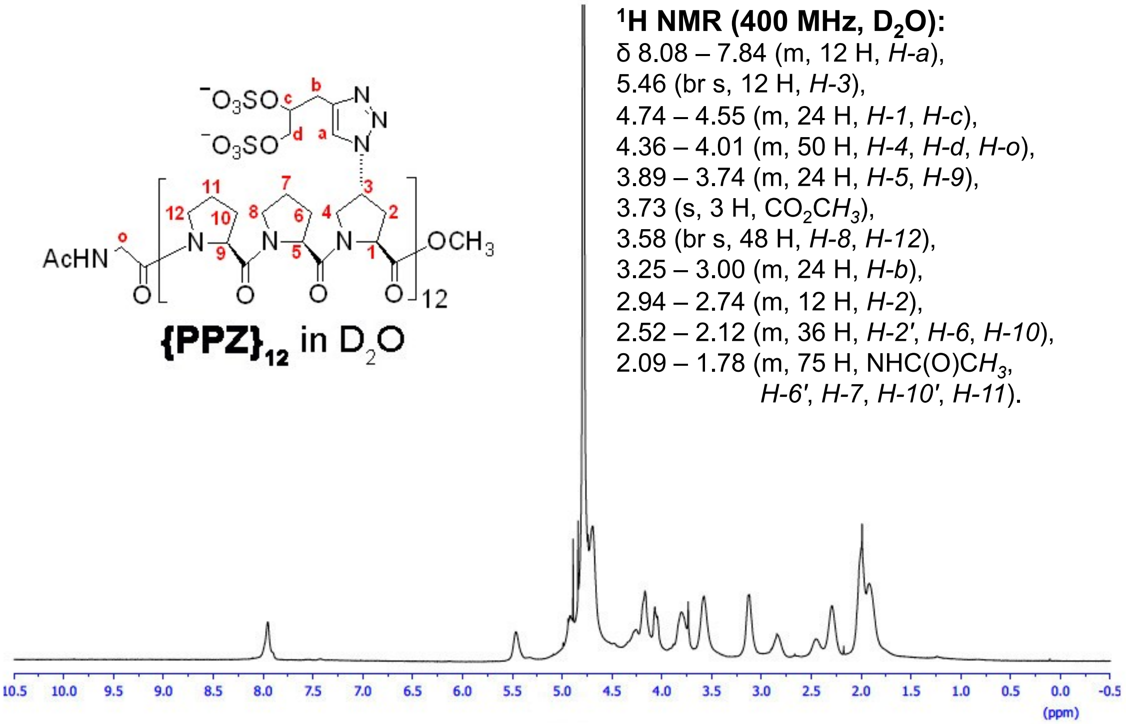

Fig. S11 ^{1}H NMR spectra of **{PPZ}$_{12}$**.

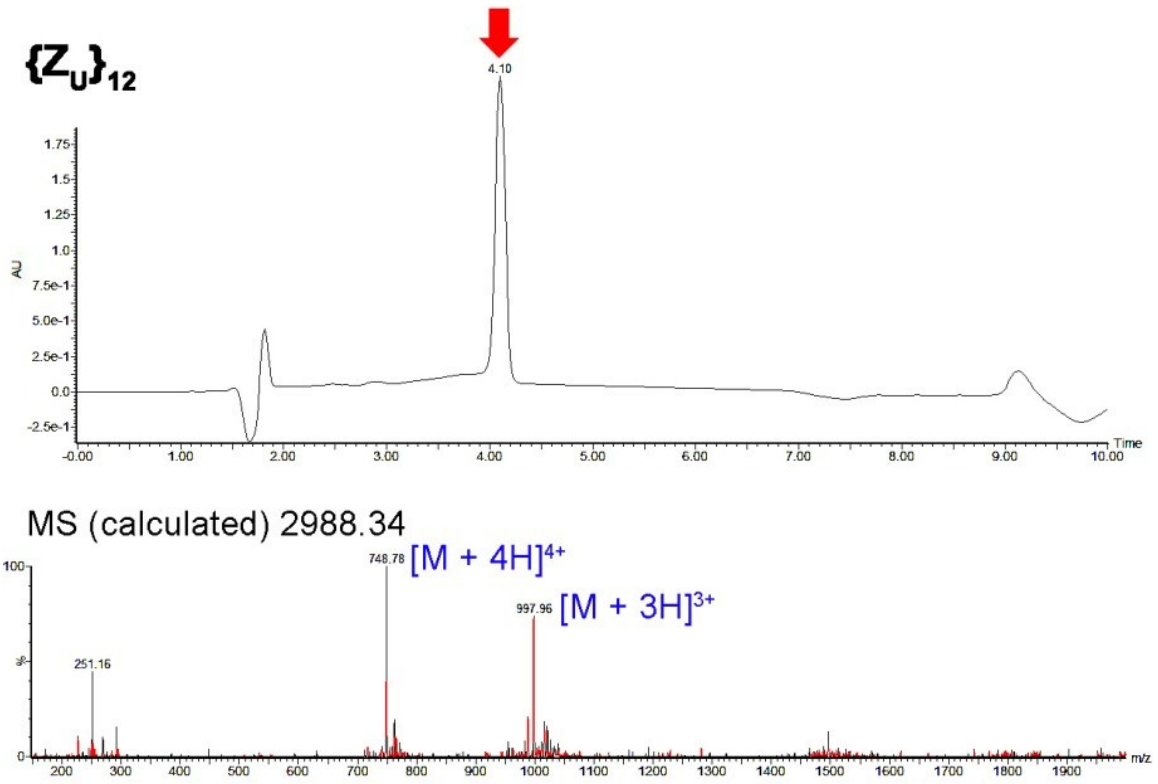

Fig. S12 Analytical HPLC traces (top) and ESI mass data (bottom) of **$\{Z_U\}_{12}$**.

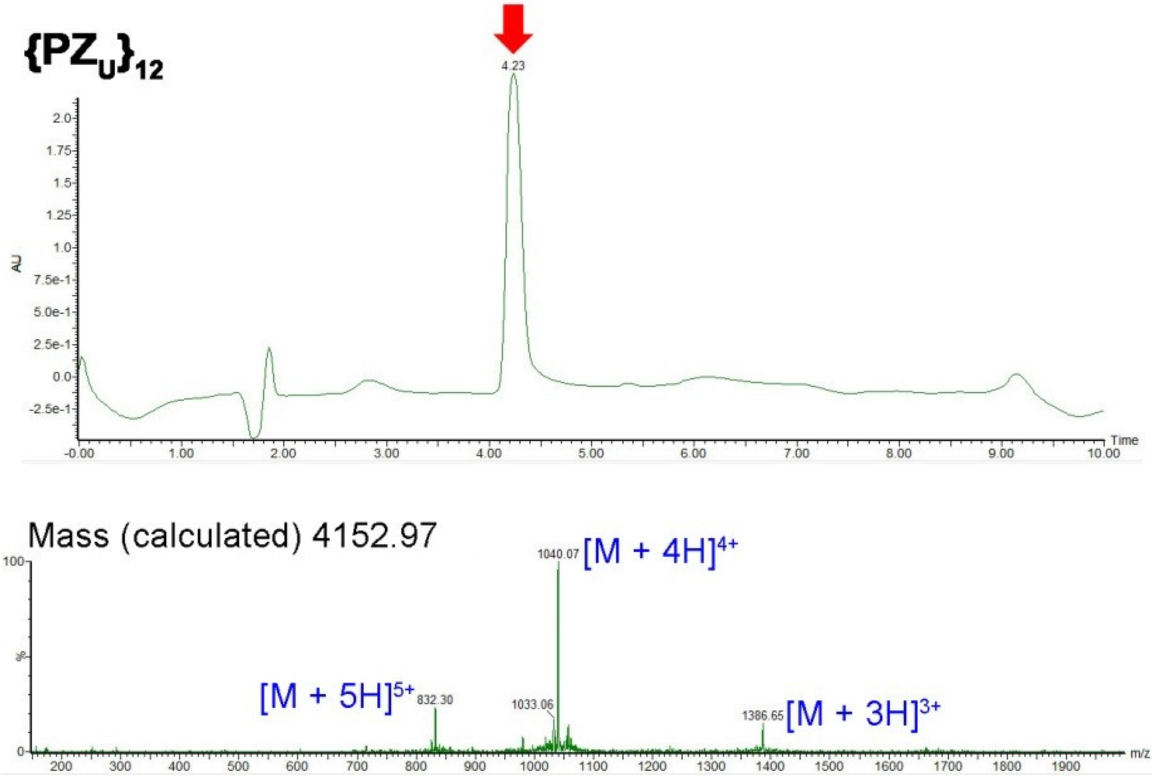

Fig. S13 Analytical HPLC traces (top) and ESI mass data (bottom) of $\{\mathbf{PZ_U}\}_{12}$.

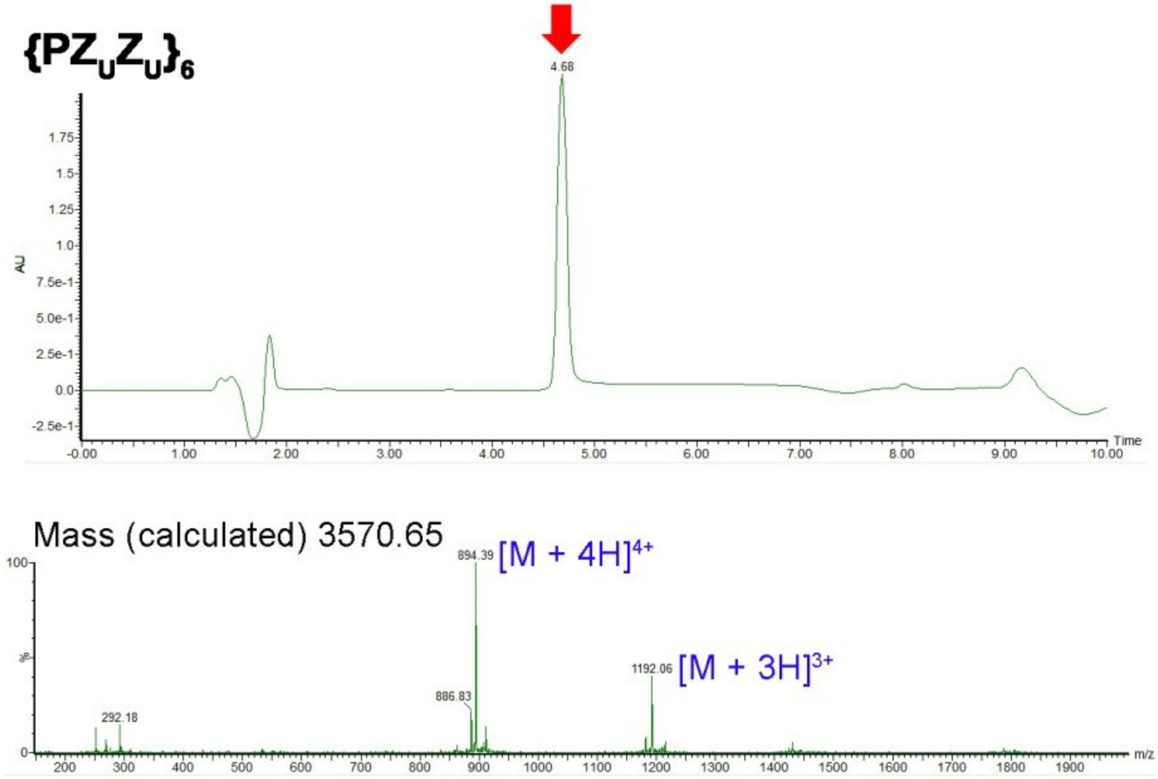

Fig. S14 Analytical HPLC traces (top) and ESI mass data (bottom) of **$\{PZ_UZ_U\}_6$**.

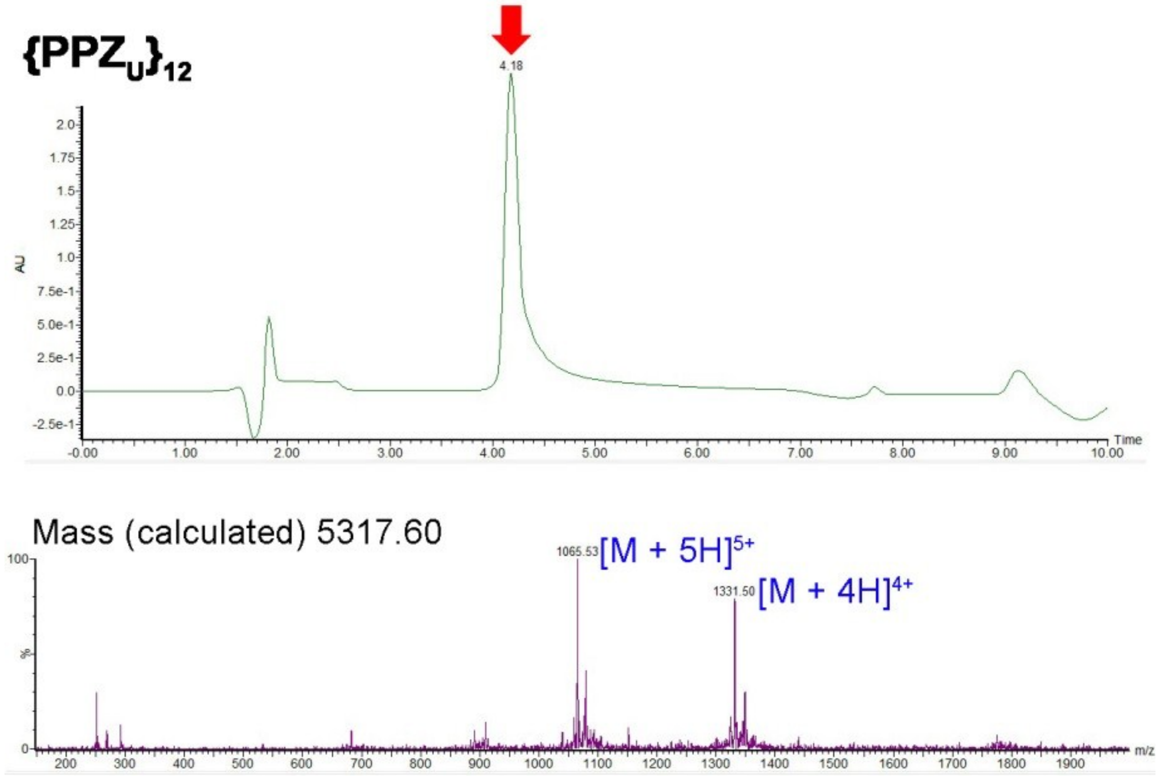

Fig. S15 Analytical HPLC traces (top) and ESI mass data (bottom) of **$\{PPZ_U\}_{12}$**.

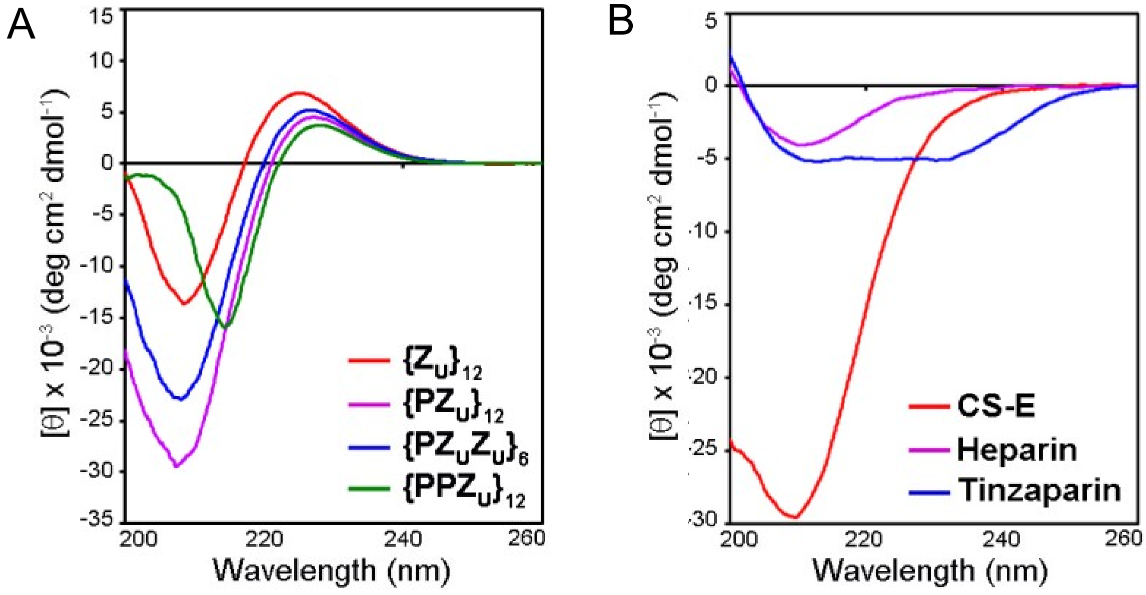

Fig. S16 (A) CD spectra of **{Z_U}$_{12}$**, {**PZ_U**}$_{12}$, **{PZ_UZ_U}$_6$** and **{PPZ_U}$_{12}$**. (B) CD spectra of CS-E, heparin and tinzaparin.

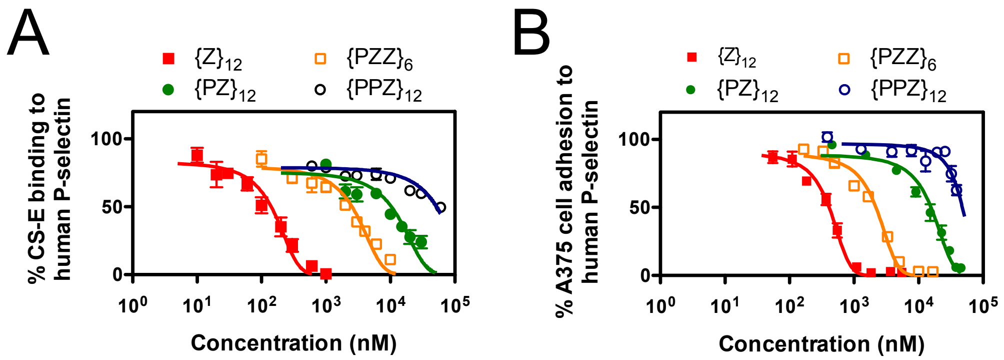

Fig. S17 (A) Inhibition of CS-E binding to human P-selectin by PGMs (n=4). (B) Inhibition of adhesion of A375 human melanoma cells to human P-selectin by PGMs (n=6).

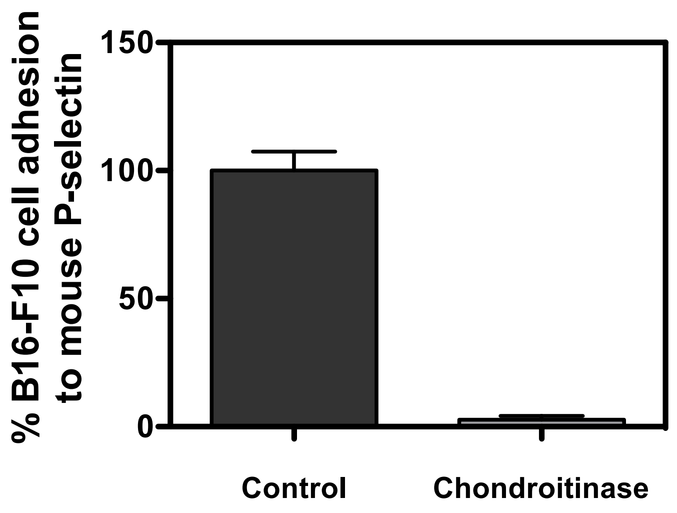

Fig. S18 Adhesion of B16-F10 Red-Fluc murine melanoma cells to mouse P-selectin upon enzymatic removal of cell surface chondroitin sulfate by chondroitinase (n=3). Cell adhesion decreased significantly compared to when no chondroitinase treatment was performed (Fig. 3A).

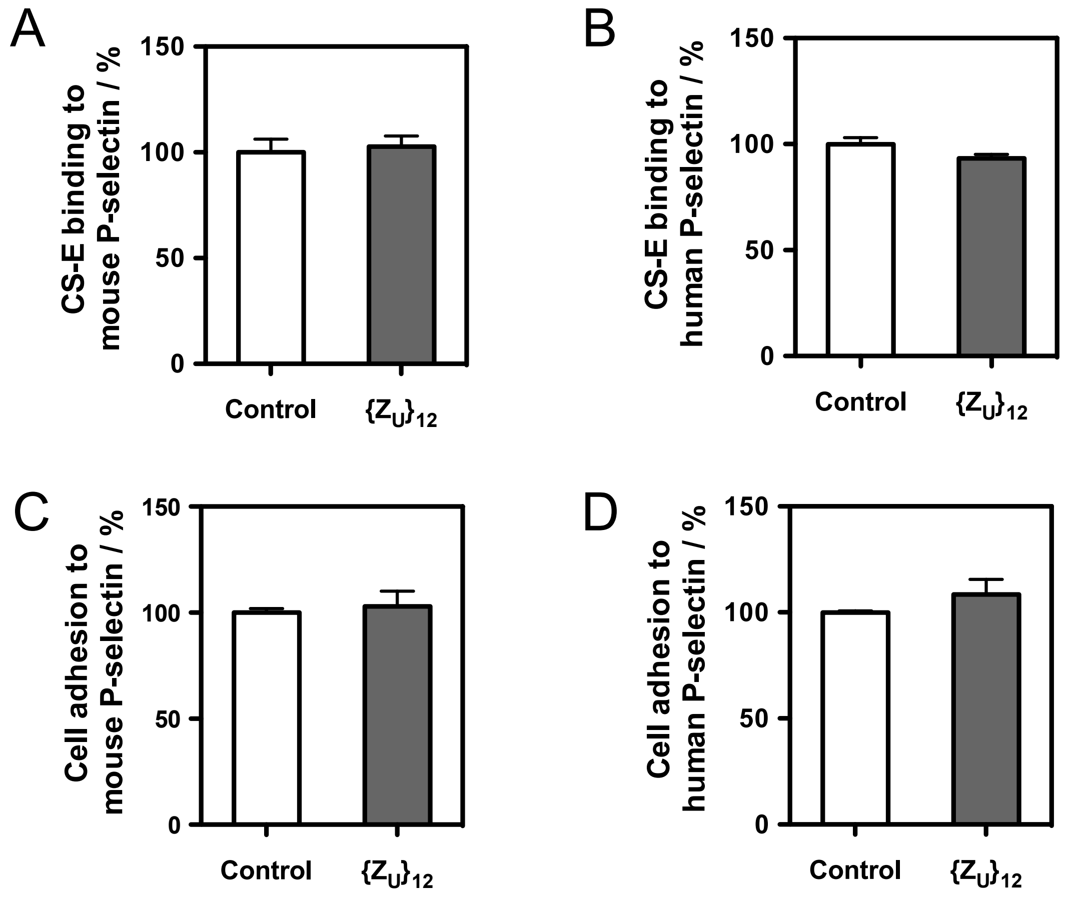

Fig. S19 (A) CS-E binding to mouse P-selectin in the presence of 1μM $\{\mathbf{Z_U}\}_{12}$ (n=6). (B) CS-E binding to human P-selectin in the presence of 1μM $\{\mathbf{Z_U}\}_{12}$ (n=6). (C) Adhesion of B16-F10 murine melanoma cells to mouse P-selectin in the presence of 10μM $\{\mathbf{Z_U}\}_{12}$ (n=6). (D) Adhesion of A375 human melanoma cells to human P-selectin in the presence of 10μM $\{\mathbf{Z_U}\}_{12}$ (n=6).

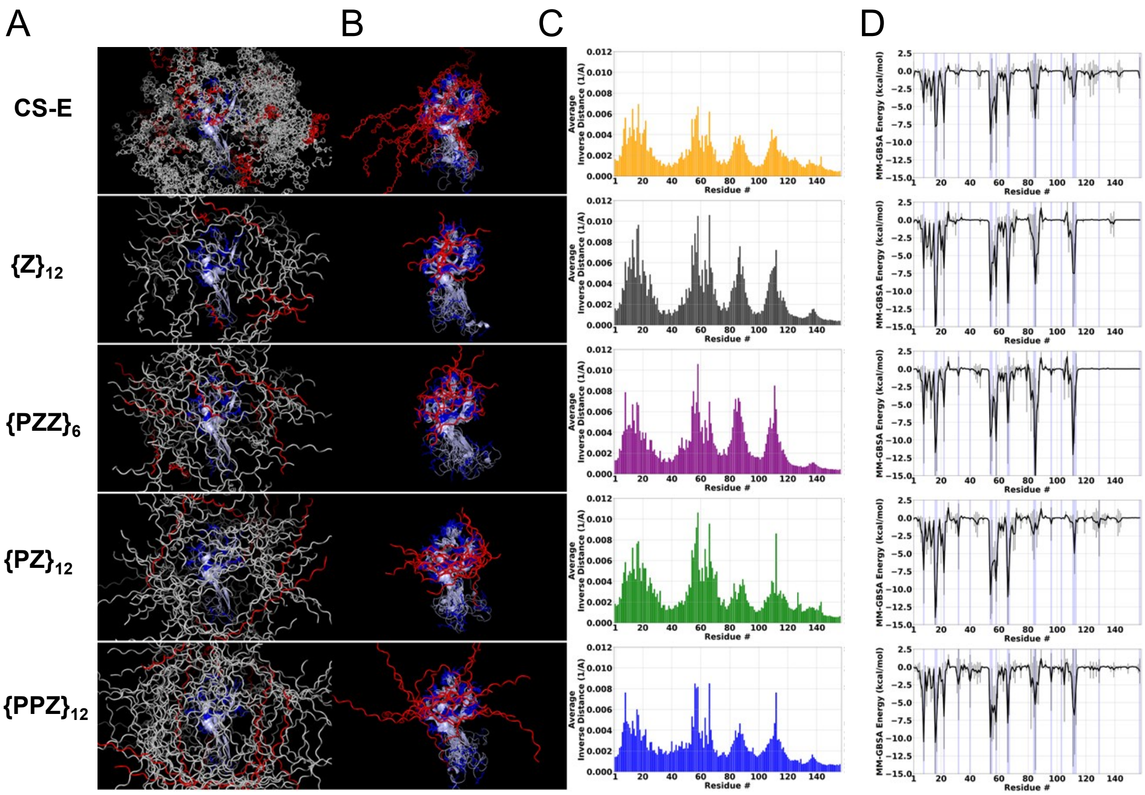

Fig. S20 All PGM structures are shown as cartoon representation. (A) 100 independent starting geometries showing the initial random placement of CS-E and PGMs around human P-selectin (blue) at the center of a 7x7x7 nm cubic box. Initial positions leading to the top 10 final poses (as ranked by MM-GBSA analyses) and the other 90 final poses are shown in red and grey respectively. (B) Human P-selectin (blue) and the top 10 final poses (red) as ranked by MM-GBSA analyses after 10 ns of implicit solvent molecular dynamics simulations, run with pmemd using igb=5, mbondi2 radii and a salt concentration of 0.15 M. (C) Plots showing average closeness of contact between top 10 final poses of CS-E/PGMs and individual residues on human P-selectin. (D) Decomposition of average MM-GBSA interaction energies along individual residues on human P-selectin for top 10 final poses of CS-E and PGMs. Blue lines denote basic amino acid residues.

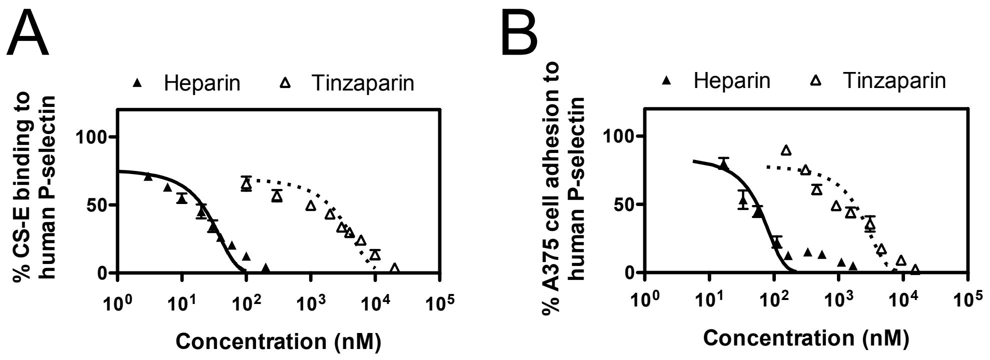

Fig. S21 (A) Inhibition of CS-E binding to human P-selectin by heparin and tinzaparin (n=4). (B) Inhibition of adhesion of A375 human melanoma cells to human P-selectin by saccharide-based variants of PGMs (n=6).

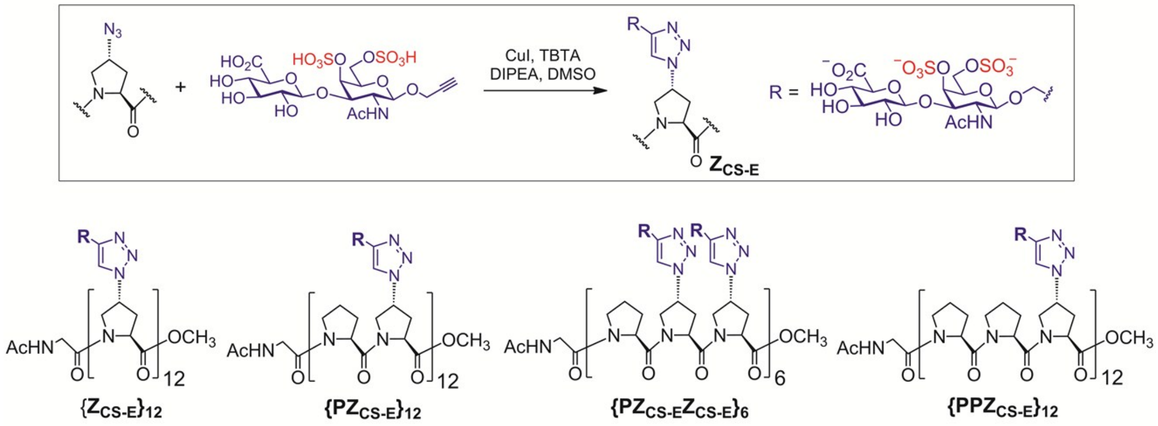

Fig. S22 Click reaction for the synthesis of saccharide-based PGMs **$\{Z_{CS-E}\}_{12}$**, **$\{PZ_{CS-E}\}_{12}$**, **$\{PZ_{CS-E}Z_{CS-E}\}_6$**, and **$\{PPZ_{CS-E}\}_{12}$**, all of which bear a total of 12 CS-E disaccharides.

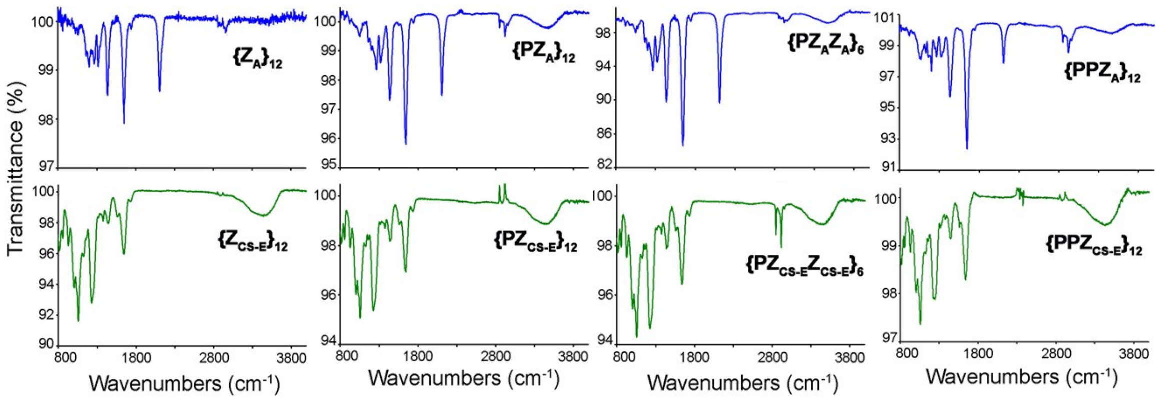

Fig. S23 FT-IR spectra of (blue) PGM precursors **$\{Z_A\}_{12}$**, **$\{PZ_A\}_{12}$**, **$\{PZ_AZ_A\}_6$** and **$\{PPZ_A\}_{12}$** and (green) the corresponding final saccharide-based PGMs **$\{Z_{CS\text{-}E}\}_{12}$**, **$\{PZ_{CS\text{-}E}\}_{12}$**, **$\{PZ_{CS\text{-}E}Z_{CS\text{-}E}\}_6$** and **$\{PPZ_{CS\text{-}E}\}_{12}$** after cycloaddition of CS-E disaccharides.

^{1}H NMR (400 MHz, D_2O):
δ 8.22 – 8.03 (m, 12 H, *H-a*),
5.48 (bs, 12 H, *H-3*),
5.08 – 4.90 (m, 24 H, *H-1*, *H-f*),
4.66 – 4.52 (m, 12 H, *H-c*),
4.52 – 4.42 (m, 12 H, *H-i*),
4.42 – 4.10 (m, 74 H, *H-4*, *H-b*, *H-h*, *H-o*),
4.10 – 3.93 (m, 36 H, *H-d*, *H-e*, *H-g*),
3.93 – 3.79 (m, 12 H, *H-n*),
3.76 (s, 3 H, CO_2CH_3),
3.55 – 3.40 (m, 24 H, *H-k*, *H-m*),
3.30 (t, *J* = 8.0 Hz, 12 H, *H-j*),
2.94 (bs, 12 H, *H-2*),
2.73 – 2.38 (m, 12 H, *H-2'*),
2.00 – 1.74 (m, 39 H, 13 NHC(O)CH_3)

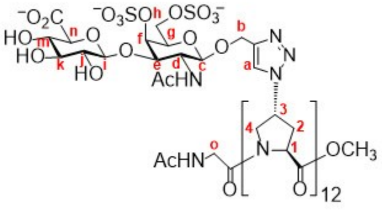

{Z$_{CS-E}$}$_{12}$ in D_2O

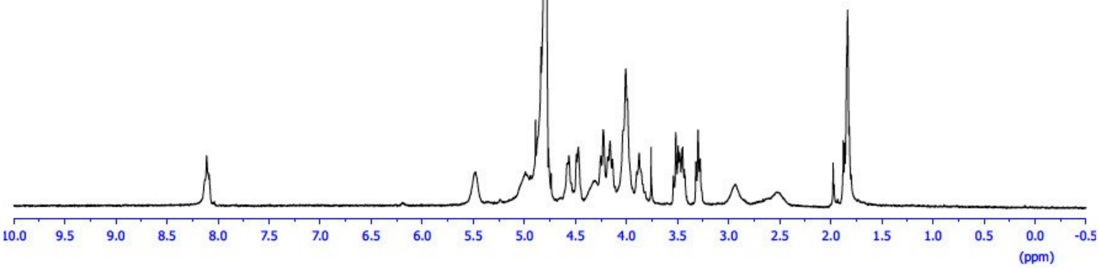

Fig. S24 ^{1}H NMR spectra of **{Z$_{CS-E}$}$_{12}$**.

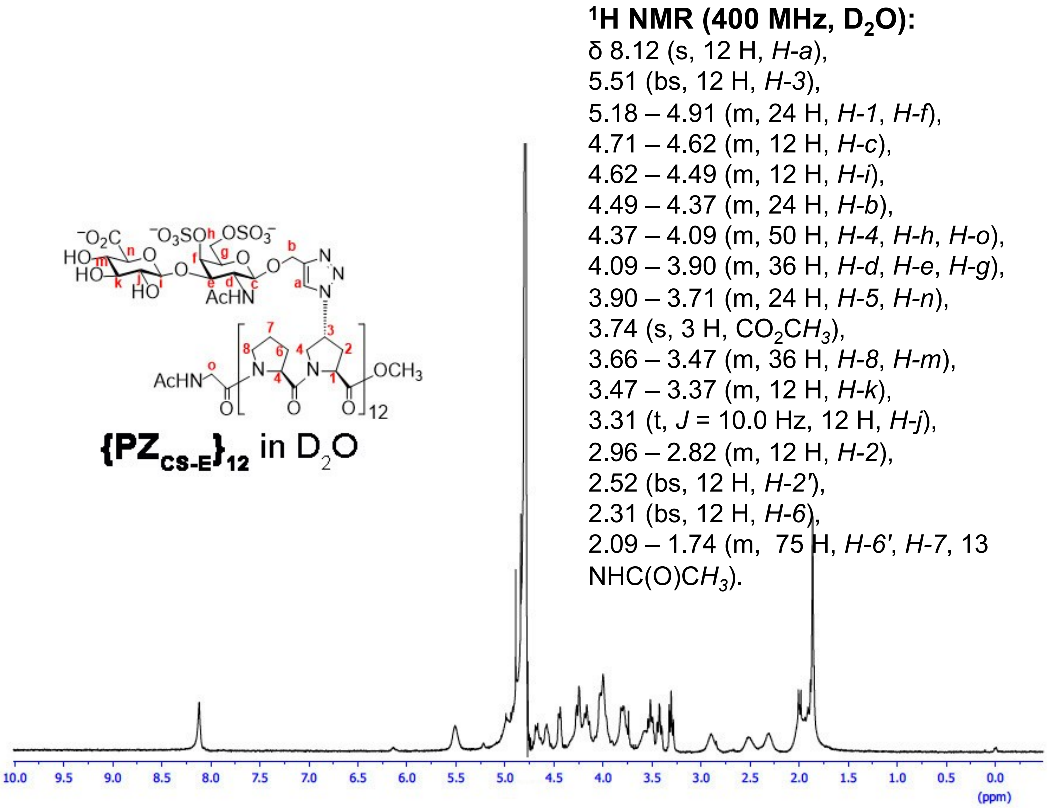

Fig. S25 ^{1}H NMR spectra of **{PZ$_{CS-E}$}$_{12}$**.

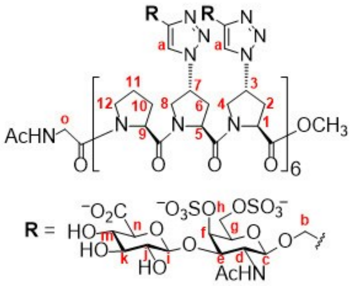

{PZ$_{CS\text{-}E}$Z$_{CS\text{-}E}$}$_6$ in D_2O

^{1}H NMR (400 MHz, D_2O):
δ 8.20 – 7.98 (m, 12 H, *H-a*),
5.57 – 5.33 (m, 12 H, *H-3*, *H-7*),
5.06 – 4.90 (m, 24 H, *H-1*, *H-5*, *H-f*),
4.74 – 4.65 (m, 12 H, *H-c*),
4.65 – 4.52 (m, 12 H, *H-i*),
4.52 – 4.40 (m, 24 H, *H-b*),
4.40 – 4.09 (m, 50 H, *H-4*, *H-8*, *H-h*, *H-o*),
4.09 – 3.93 (m, 36 H, *H-d*, *H-e*, *H-g*),
3.93 – 3.71 (m, 18 H, *H-9*, *H-n*),
3.76 (s, 3 H, CO_2CH_3),
3.64 – 3.38 (m, 36 H, *H-12*, *H-k*, *H-m*),
3.31 (t, J = 8.0 Hz, 12 H, *H-j*),
3.02 – 2.79 (m, 12 H, *H-2*, *H-6*),
2.60 – 2.22 (m, 12 H, *H-2'*, *H-6'*),
2.12 – 1.75 (m, 63 H, *H-10*, *H-11*, 13 NHC(O)CH_3).

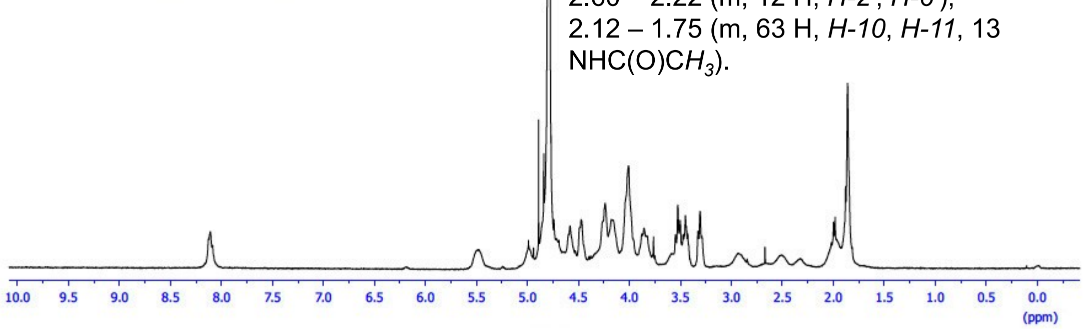

Fig. S26 ^{1}H NMR spectra of **{PZ$_{CS\text{-}E}$Z$_{CS\text{-}E}$}$_6$**.

^{1}H NMR (400 MHz, D_2O):
δ 8.09 (s, 12 H, *H-a*),
5.49 (bs, 12 H, *H-3*),
5.08 – 4.90 (m, 24 H, *H-1*, *H-f*),
4.74 – 4.64 (m, 12 H, *H-c*),
4.64 – 4.51 (m, 12 H, *H-i*),
4.51 – 4.35 (m, 24 H, *H-b*),
4.35 – 4.09 (m, 50 H, *H-4*, *H-h*, *H-o*),
4.09 – 3.92 (m, 36 H, *H-d*, *H-e*, *H-g*),
3.92 – 3.62 (m, 36 H, *H-5*, *H-9*, *H-n*),
3.74 (s, 3 H, CO_2CH_3),
3.59 – 3.52 (m, 36 H, *H-8*, *H-m*),
3.52 – 3.37 (m, 36 H, *H-12*, *H-k*),
3.29 (t, *J* = 10.0 Hz, 12 H, *H-j*),
2.88 (bs, 12 H, *H-2*),
2.46 (bs, 12 H, *H-2′*),
2.30 (bs, 24 H, *H-6*, *H-10*),
2.11 – 1.71 (m, 111 H, *H-6′*, *H-7*, *H-10′*, *H-11*, 13 $NHC(O)CH_3$).

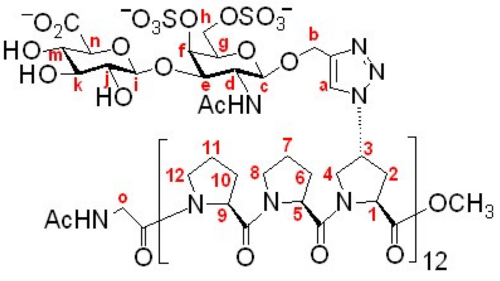

{PPZ$_{CS-E}$}$_{12}$ in D_2O

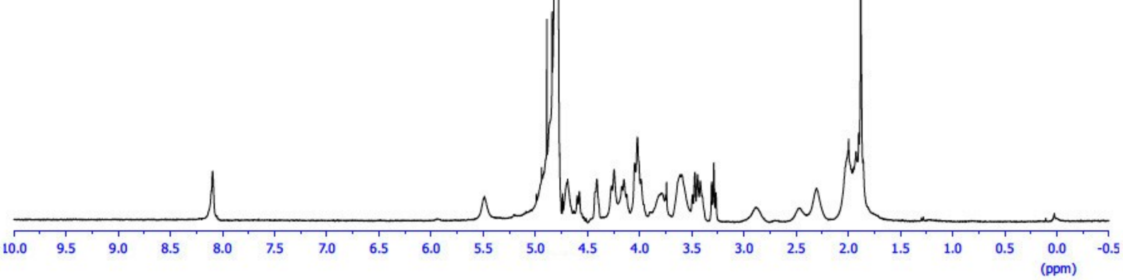

Fig. S27 ^{1}H NMR spectra of **{PPZ$_{CS-E}$}$_{12}$**.

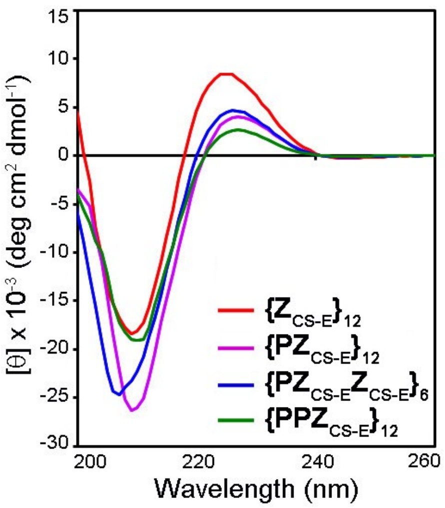

Fig. S28 CD spectra of **{Z$_{\mathbf{CS\text{-}E}}$}$_{\mathbf{12}}$**, **{PZ$_{\mathbf{CS\text{-}E}}$}$_{\mathbf{12}}$**, **{PZ$_{\mathbf{CS\text{-}E}}$Z$_{\mathbf{CS\text{-}E}}$}$_{\mathbf{6}}$** and **{PPZ$_{\mathbf{CS\text{-}E}}$}$_{\mathbf{12}}$**.

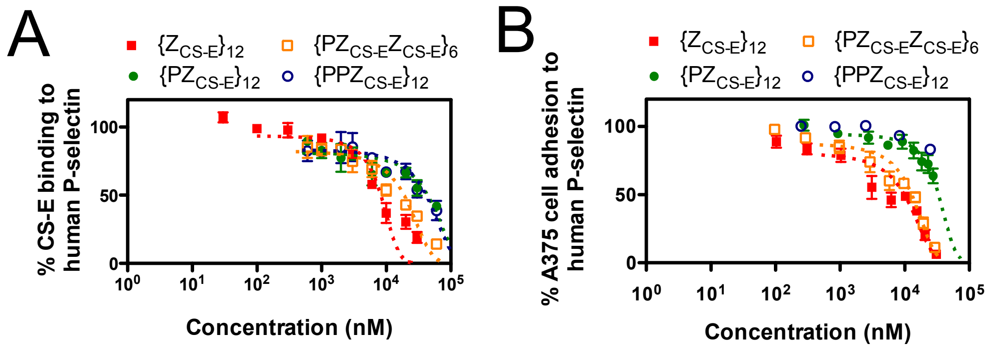

Fig. S29 (A) Inhibition of CS-E binding to human P-selectin by saccharide-based variants of PGMs (n=4). (B) Inhibition of adhesion of A375 human melanoma cells to human P-selectin by saccharide-based variants of PGMs (n=6).

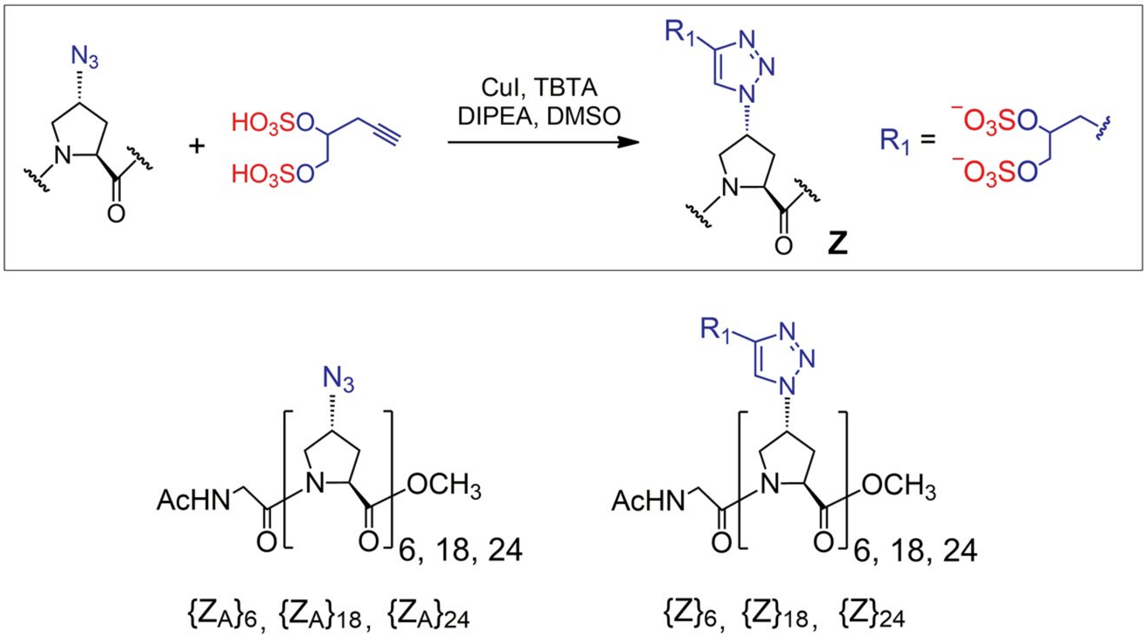

Fig. S30 Structures of PGM precursors bearing 6, 18, and 24 Z_A units and PGMs bearing 6, 18, and 24 Z units.

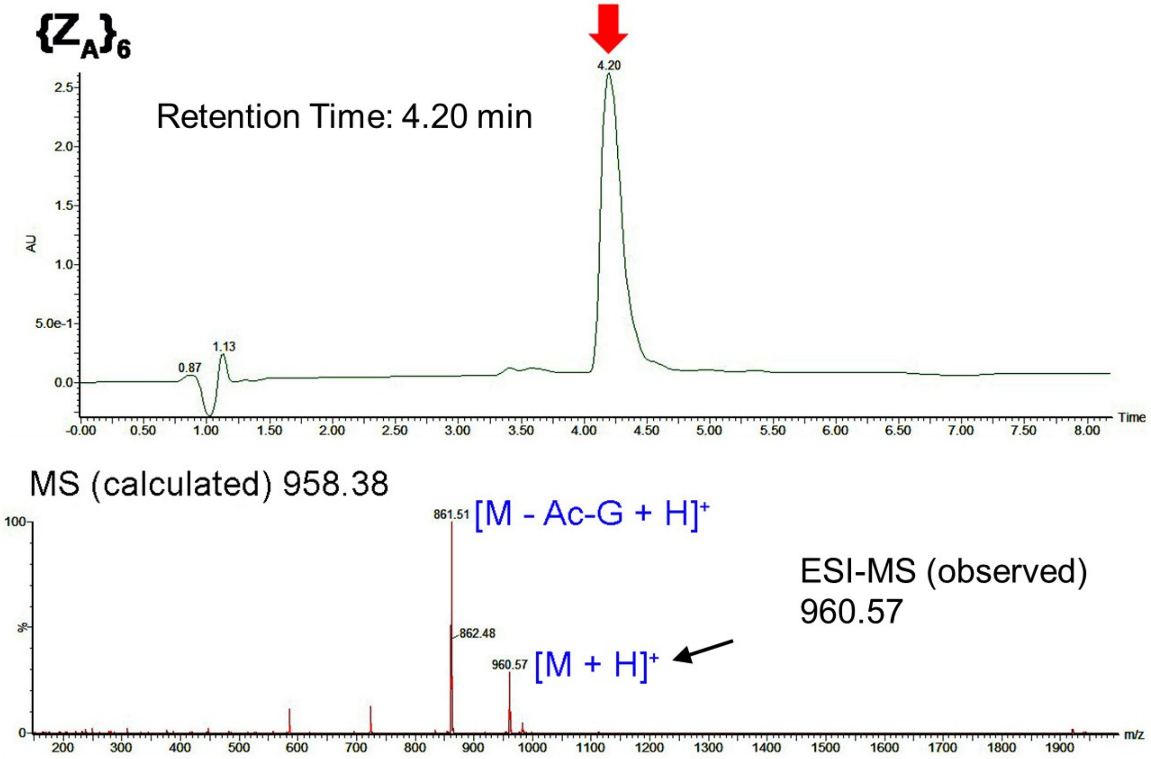

Fig. S31 Analytical HPLC traces (top) and ESI mass data (bottom) of **$\{Z_A\}_6$**.

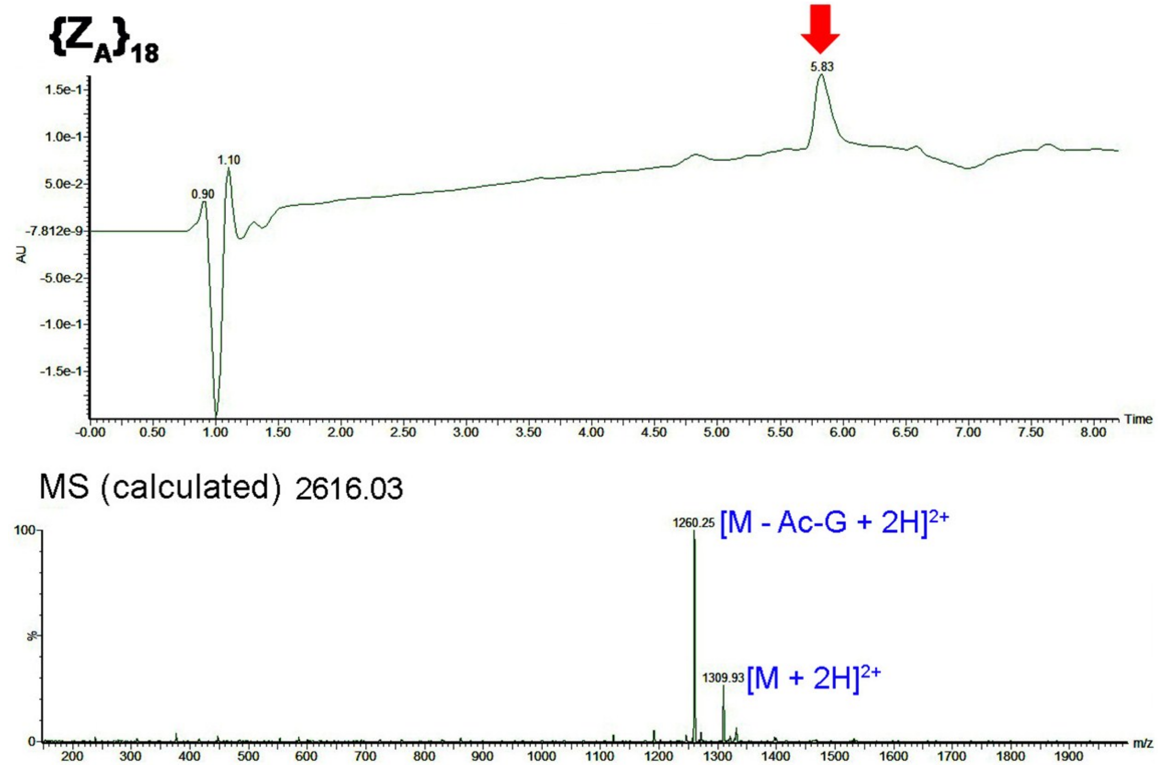

Fig. S32 Analytical HPLC traces (top) and ESI mass data (bottom) of **$\{Z_A\}_{18}$**.

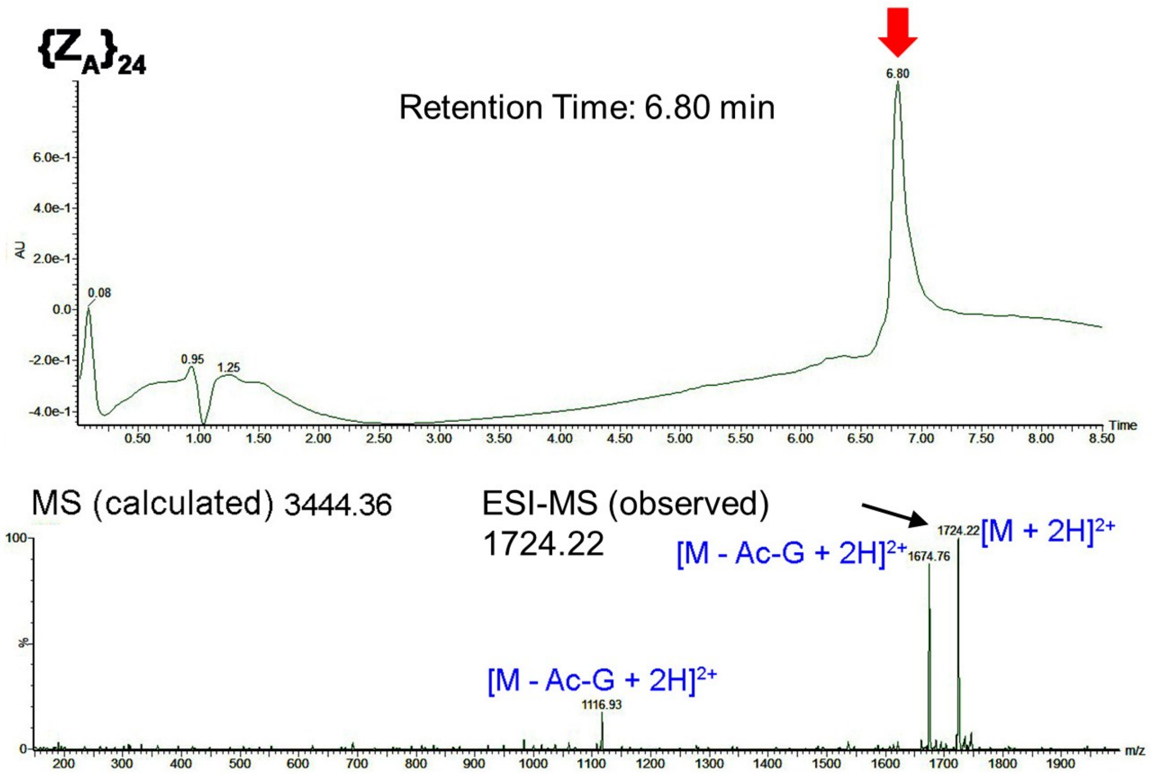

Fig. S33 Analytical HPLC traces (top) and ESI mass data (bottom) of **$\{Z_A\}_{24}$**.

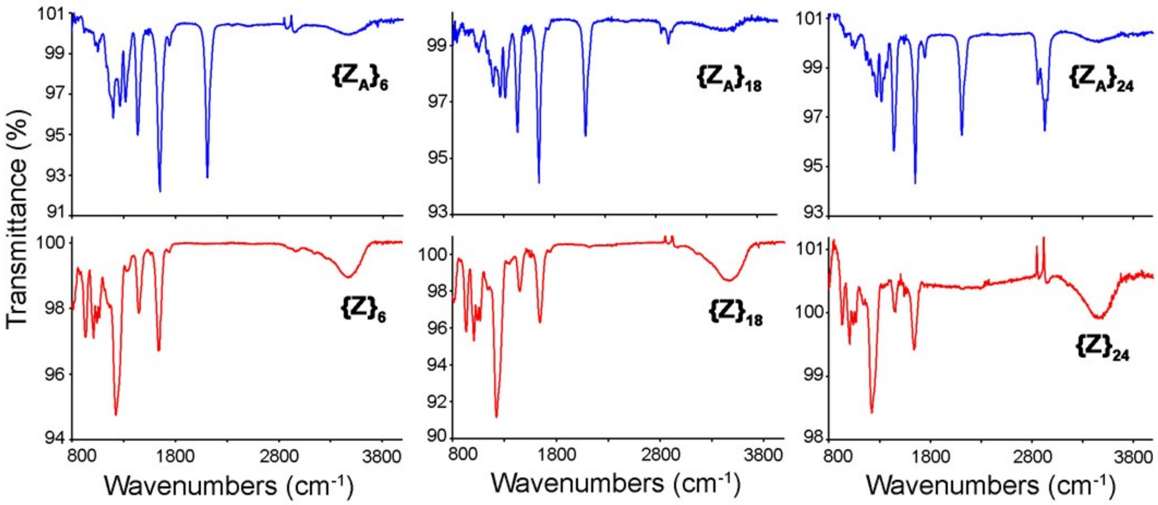

Fig. S34 FT-IR spectra of (blue) PGM precursors **$\{Z_A\}_6$**, **$\{Z_A\}_{18}$** and **$\{Z_A\}_{24}$** and (red) the corresponding final PGMs **$\{Z\}_6$**, **$\{Z\}_{18}$** and **$\{Z\}_{24}$** after cycloaddition of sulfate moieties.

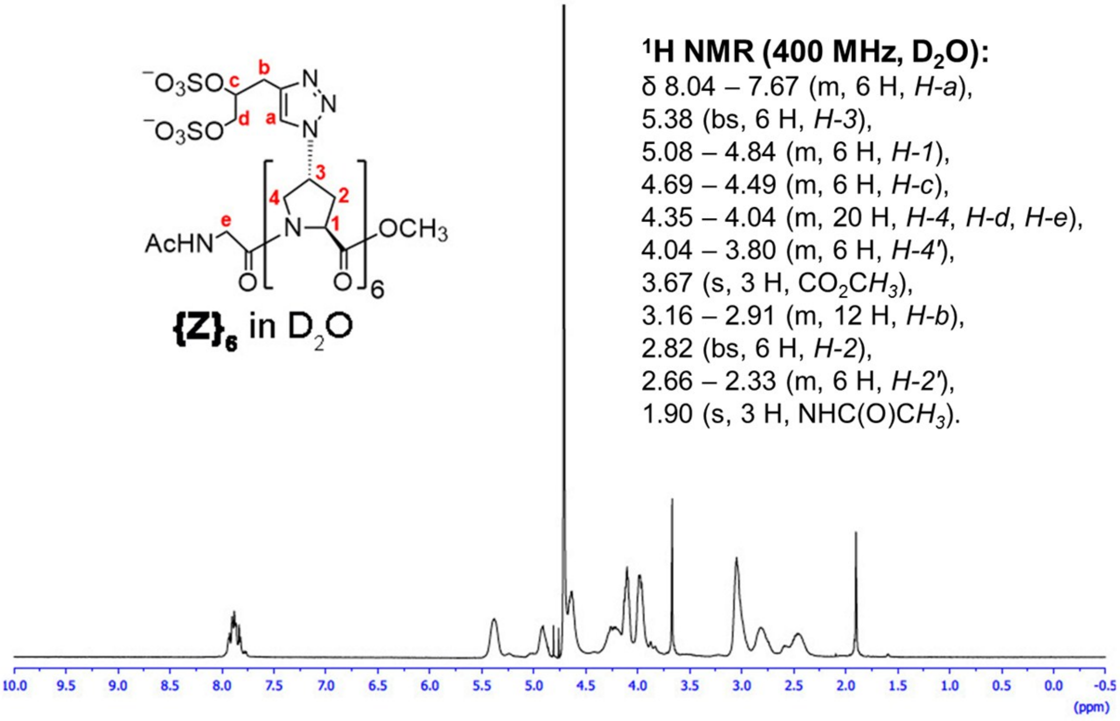

Fig. S35 ^{1}H NMR spectra of **$\{Z\}_6$**.

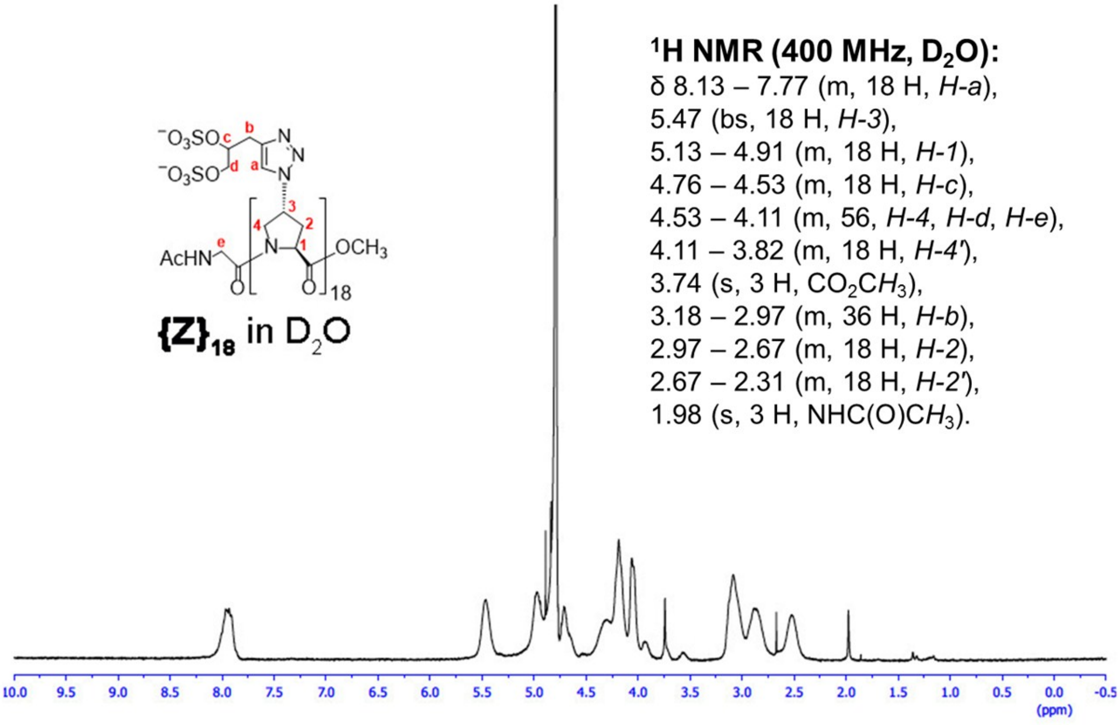

Fig. S36 ^{1}H NMR spectra of **$\{Z\}_{18}$**.

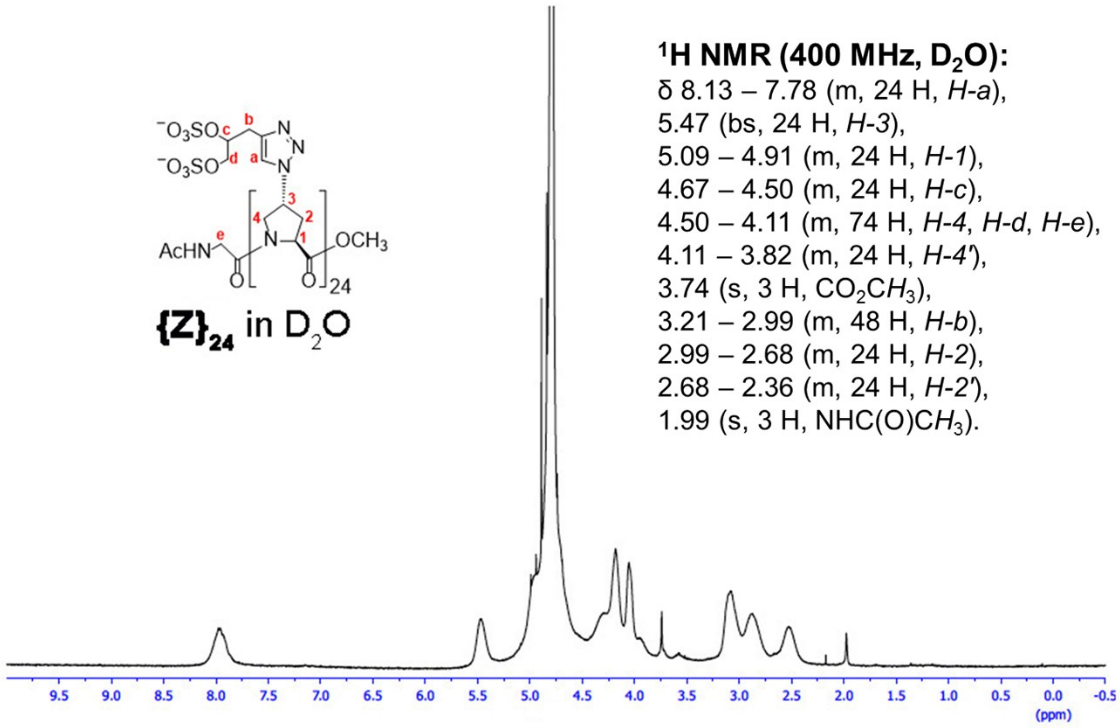

Fig. S37 ^{1}H NMR spectra of $\{\mathbf{Z}\}_{24}$.

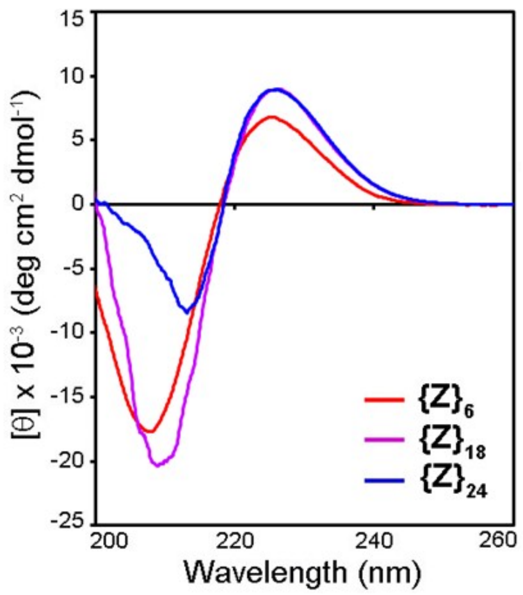

Fig. S38 CD spectra of PGMs **{Z}$_6$**, **{Z}$_{18}$** and **{Z}$_{24}$**.

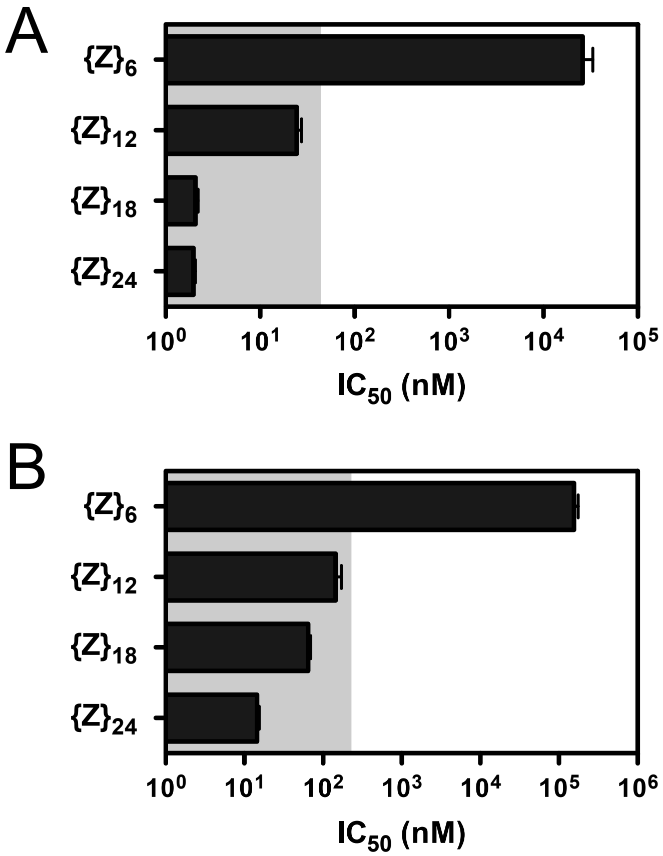

Fig. S39 IC_{50} values for **$\{Z\}_6$**, **$\{Z\}_{12}$**, **$\{Z\}_{18}$** and **$\{Z\}_{24}$** on inhibiting CS-E binding to (A) mouse (n=4) and (B) human P-selectin (n=4).

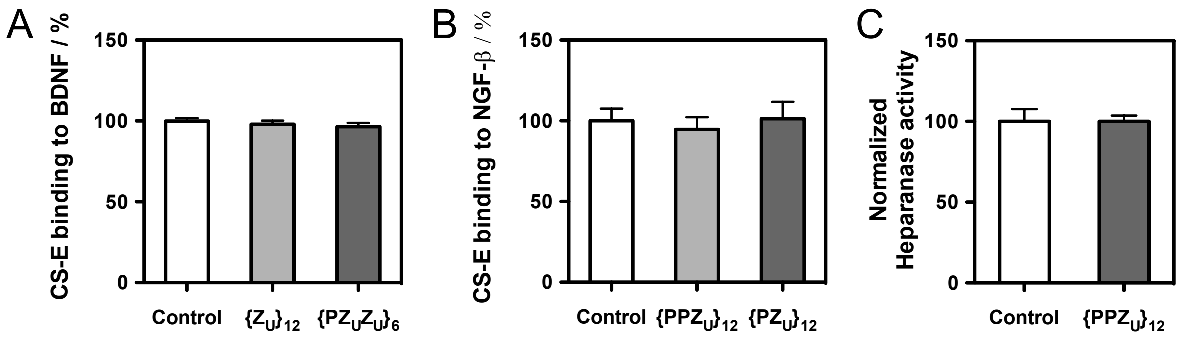

Fig. S40 (A) CS-E binding to human BDNF in the presence of 10μM **{Z$_U$}$_{12}$** and 10μM **{PZ$_U$Z$_U$}$_6$** (n=5). (B) CS-E binding to human NGF-β in the presence of 15μM **{Z$_U$}$_{12}$** and 15μM **{PZ$_U$}$_{12}$** (n=9). (C) Normalized heparinase activity in the presence of 6μM **{PPZ$_U$}$_{12}$** (n=6).

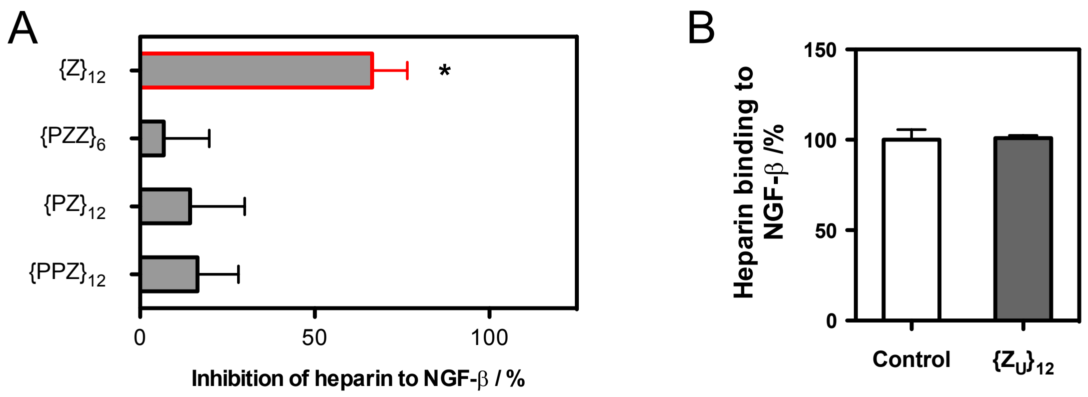

Fig. S41 (A) Inhibition of heparin binding to human NGF-β by 10μM PGMs (n=6). (B) Heparin binding to human NGF-β in the presence of 10μM **$\{Z_U\}_{12}$** (n=5).

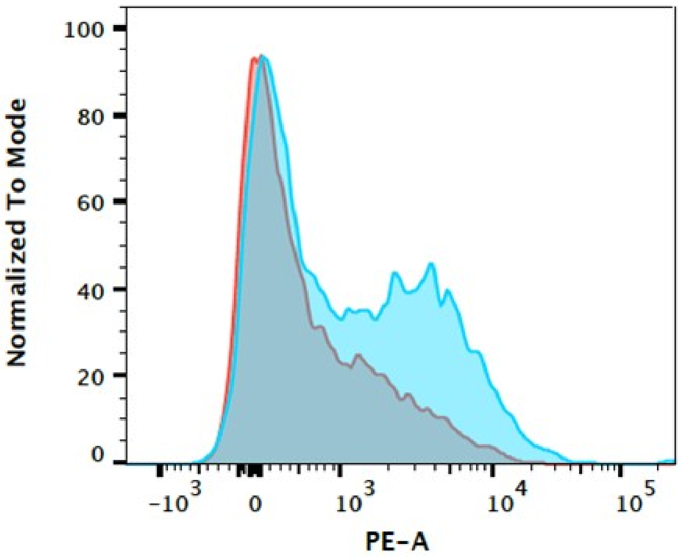

Figure S42 Representative flow cytometry histogram showing resting (red) and activated (blue) platelets stained with PE-Anti-mouse P-selectin.

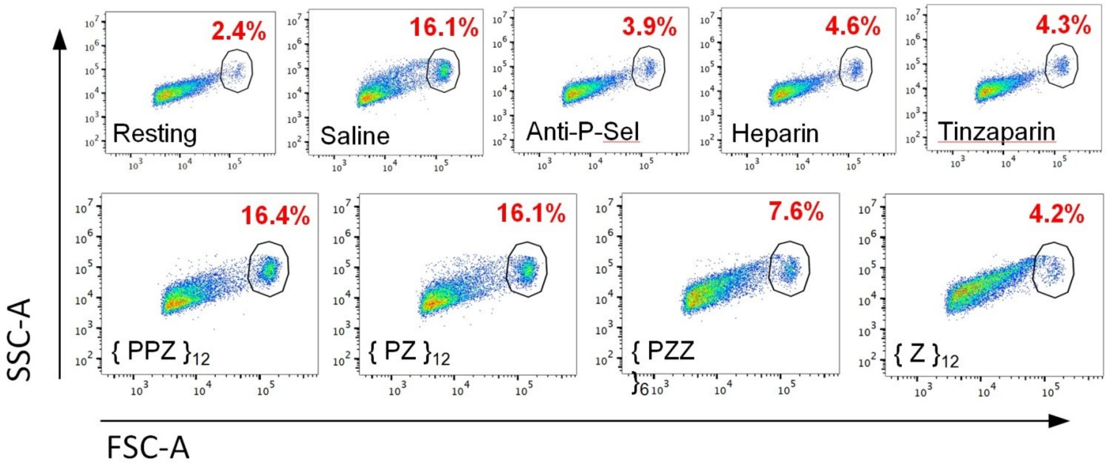

Fig. S43 Representative flow cytometry histogram showing resting (red) and activated (blue) platelets stained with PE-Anti-mouse P-selectin.

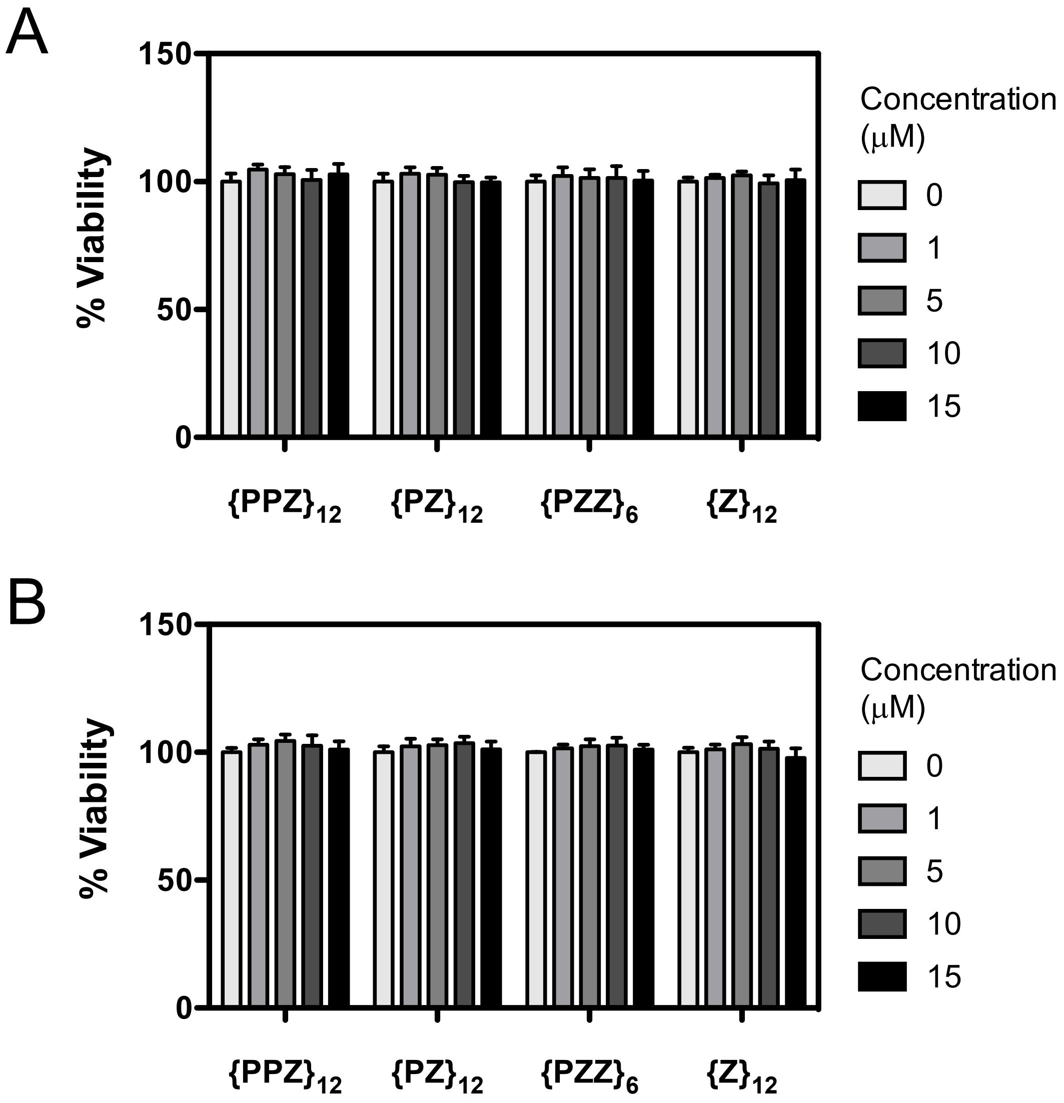

Figure S44. Viability of B16-F10 Red-Fluc murine melanoma cells upon (A) 2 hr and (B) 24 hr exposure to PGMs (n=6).

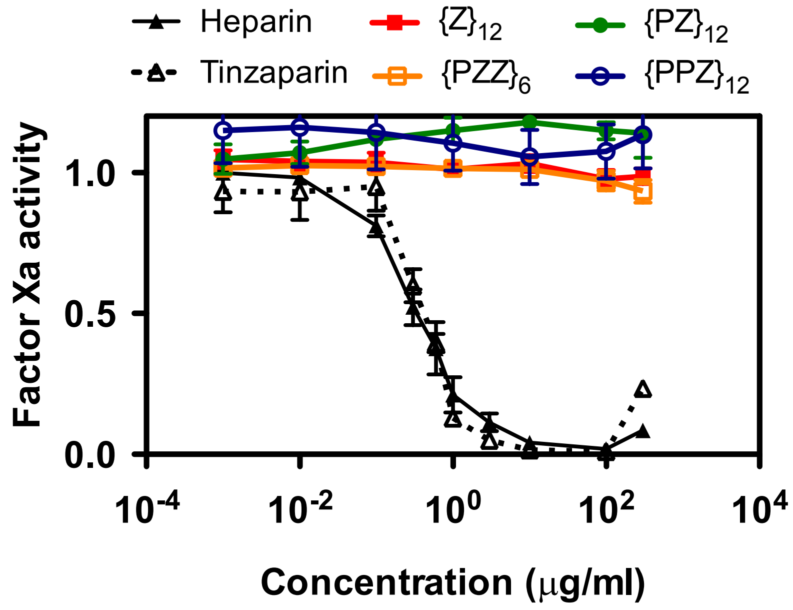

Fig. S45 Activity of blood coagulation factor Xa with varying concentrations of heparin, tinzaparin, **{PPZ}$_{12}$**, **{PZ}$_{12}$**, **{PZZ}$_6$** and **{Z}$_{12}$** (n=4).

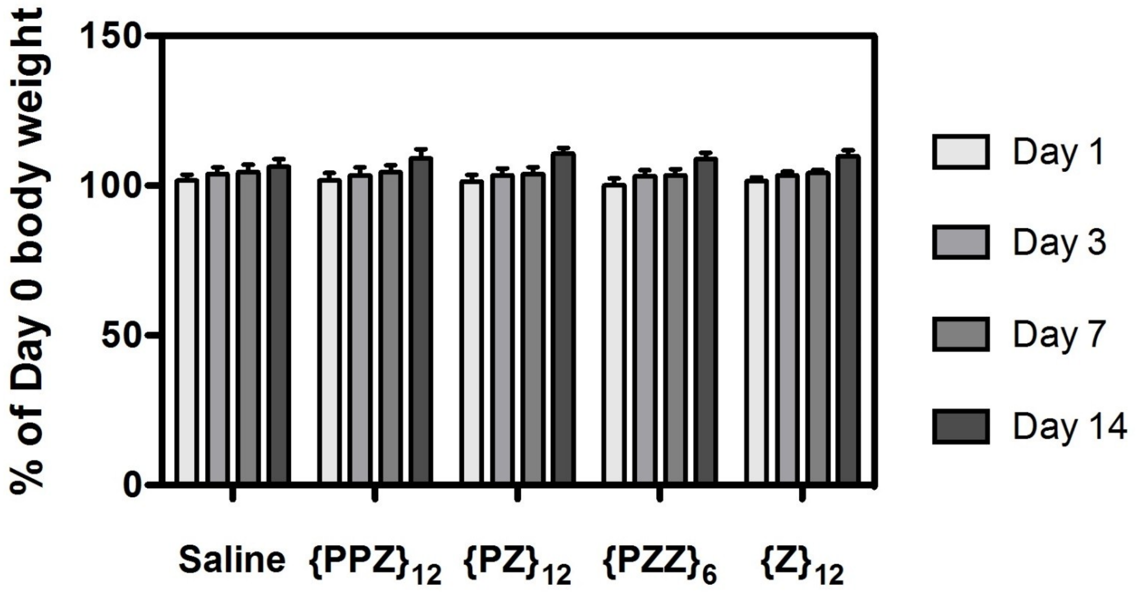

Fig. S46 Normalized body weight of mice at varying time points after injection of saline and PGMs (n=13 mice).

	IC_{50} (nM)	IC_{50} (µg/ml)
**$\{PPZ\}_{12}$**	28747.8 ± 1955.5	223.37 ± 15.19
**$\{PZ\}_{12}$**	3027.8 ± 348.0	20.00 ± 2.30
**$\{PZZ\}_{6}$**	325.5 ± 38.0	1.96 ± 0.23
**$\{Z\}_{12}$**	24.4 ± 3.0	0.13 ± 0.02
Heparin	21.6 ± 2.4	0.39 ± 0.04
Tinzaparin	1339.6 ± 121.6	8.71 ± 0.79
**$\{PPZ_{CS\text{-}E}\}_{12}$**	23583.5 ± 4824.5	284.34 ± 58.17
**$\{PZ_{CS\text{-}E}\}_{12}$**	14889.1 ± 1263.3	162.16 ± 13.76
**$\{PZ_{CS\text{-}E}Z_{CS\text{-}E}\}_{6}$**	2044.0 ± 203.1	21.07 ± 2.09
**$\{Z_{CS\text{-}E}\}_{12}$**	892.0 ± 265.5	8.68 ± 2.58

Table S1 IC_{50} values for PGMs, heparin and tinzaparin on inhibiting the binding of CS-E to mouse P-selectin. (n=4, mean ± SEM)

	IC_{50} (nM)			IC_{50} (μg/ml)		
**$\{PPZ\}_{12}$**	52019.3	±	726.3	404.18	±	5.64
**$\{PZ\}_{12}$**	10693.9	±	1395.0	70.63	±	9.21
**$\{PZZ\}_{6}$**	2521.4	±	176.2	15.18	±	1.06
**$\{Z\}_{12}$**	144.8	±	26.9	0.79	±	0.15
Heparin	20.7	±	2.3	0.37	±	0.04
Tinzaparin	1986.9	±	188.7	12.91	±	1.23
**$\{PPZ_{CS-E}\}_{12}$**	41898.4	±	2601.7	505.16	±	31.37
**$\{PZ_{CS-E}\}_{12}$**	43448.7	±	1715.9	473.22	±	18.69
**$\{PZ_{CS-E}Z_{CS-E}\}_{6}$**	18654.0	±	1618.7	192.30	±	16.69
**$\{Z_{CS-E}\}_{12}$**	11110.2	±	2555.0	108.06	±	24.85

Table S2 IC_{50} values for PGMs, heparin and tinzaparin on inhibiting the binding of CS-E to human P-selectin. (n=4, mean ± SEM)

	IC_{50} (nM)	IC_{50} (µg/ml)
**$\{PPZ\}_{12}$**	28556.5 ± 2670.6	221.88 ± 20.75
**$\{PZ\}_{12}$**	2672.2 ± 108.0	17.65 ± 0.71
**$\{PZZ\}_{6}$**	266.2 ± 32.9	1.60 ± 0.20
**$\{Z\}_{12}$**	33.0 ± 2.8	0.18 ± 0.02
Heparin	26.9 ± 2.1	0.48 ± 0.04
Tinzaparin	979.6 ± 65.5	6.37 ± 0.43
**$\{PPZ_{CS\text{-}E}\}_{12}$**	38554.2 ± 2927.3	464.84 ± 35.29
**$\{PZ_{CS\text{-}E}\}_{12}$**	3161.5 ± 274.2	34.43 ± 2.99
**$\{PZ_{CS\text{-}E}Z_{CS\text{-}E}\}_{6}$**	867.5 ± 48.0	8.94 ± 0.50
**$\{Z_{CS\text{-}E}\}_{12}$**	304.4 ± 16.6	2.96 ± 0.16

Table S3 IC_{50} values for PGMs, heparin and tinzaparin on inhibiting the adhesion of B16-F10 Red-Fluc murine melanoma cells to mouse P-selectin**.** (n=9, mean ± SEM)

	IC_{50} (nM)			IC_{50} (µg/ml)		
**$\{PPZ\}_{12}$**	45872.0	±	2077.4	356.42	±	16.14
**$\{PZ\}_{12}$**	15572.7	±	1427.9	102.85	±	9.43
**$\{PZZ\}_{6}$**	2193.1	±	84.3	13.21	±	0.51
**$\{Z\}_{12}$**	413.7	±	39.1	2.25	±	0.21
Heparin	56.4	±	7.9	1.01	±	0.14
Tinzaparin	1741.4	±	212.6	11.32	±	1.38
**$\{PPZ_{CS-E}\}_{12}$**	>>		25000*	>>		300*
**$\{PZ_{CS-E}\}_{12}$**	32148.5	±	2508.1	350.14	±	27.32
**$\{PZ_{CS-E}Z_{CS-E}\}_{6}$**	12805.0	±	1056.3	132.00	±	10.89
**$\{Z_{CS-E}\}_{12}$**	9727.2	±	935.6	94.61	±	9.1

Table S4 IC_{50} values for PGMs, heparin and tinzaparin on inhibiting the adhesion of A375 human melanoma cells to human P-selectin. * Only ~80% inhibition at 300 µg/ml, fitting did not converge to yield IC_{50}. (n=6, mean ± SEM)

	IC_{50} (nM)	IC_{50} (μg/ml)
**{Z}$_6$**	26060.5 ± 7284.5	72.582 ± 20.288
**{Z}$_{18}$**	2.1 ± 0.1	0.017 ± 0.001
**{Z}$_{24}$**	2.0 ± 0.1	0.021 ± 0.001

Table S5. IC_{50} values for **{Z}$_6$**, **{Z}$_{18}$** and **{Z}$_{24}$** on inhibiting the binding of CS-E to mouse P-selectin. (n=4, mean ± SEM)

	IC_{50} (nM)			IC_{50} (μg/ml)		
**$\{Z\}_6$**	155437.6	±	20021.0	432.914	±	55.761
**$\{Z\}_{18}$**	65.0	±	5.0	0.526	±	0.040
**$\{Z\}_{24}$**	14.5	±	1.0	0.156	±	0.010

Table S6. IC_{50} values for **$\{Z\}_6$**, **$\{Z\}_{18}$** and **$\{Z\}_{24}$** on inhibiting the binding of CS-E to human P-selectin. (n=4, mean ± SEM)

	$\{Z\}_{12}$	$\{PZZ\}_{6}$	$\{PZ\}_{12}$	$\{PPZ\}_{12}$	UP	DOWN
χ_1	25.2±11.7	21.3±16.3	19.0±18.6	16.2±20.3	-24.9±8.3	27.5±7.5
χ_2	-27.6±14.2	-23.3±20.3	-20.4±23.2	-17.4±25.5	36.7±8.6	-36.3±7.7
χ_3	20.4±14.0	17.2±18.8	14.6±21.0	12.5±23.1	-33.8±7.3	30.6±7.7
χ_4	-4.9±11.0	-4.0±12.4	-2.8±12.7	-2.3±13.6	18.6±6.6	-13.6±8.0
χ_5	-12.7±9.0	-10.9±9.8	-10.2±10.0	-8.8±10.4	3.7±7.2	-8.6±7.8

Table S7. Comparison of proline ring pucker angles for PGMs (average across the entire simulations and across all prolines ± stdev) with reference values from canonical up and down conformations.[14] All PGMs show a preference for the down pucker conformation, which is consistent with that of PPII conformation.[15]

Movie S1 Molecular dynamics simulation to visualize conformational fluctuations of heparin over 800ns.

Movie S2 Molecular dynamics simulation to visualize conformational fluctuations of CS-E over 800ns.

Movie S3 Molecular dynamics simulation to visualize conformational fluctuations of **{Z}**$_{\mathbf{12}}$ over 800ns.

Movie S4 Molecular dynamics simulation to visualize conformational fluctuations of **{PZ}**$_{\mathbf{12}}$ over 800ns.

Movie S5 Molecular dynamics simulation to visualize conformational fluctuations of **{PZZ}**$_{\mathbf{6}}$ over 800ns.

Movie S6 Molecular dynamics simulation to visualize conformational fluctuations of **{PPZ}**$_{\mathbf{12}}$ over 800ns.

Supplementary References

1 P. Liu, L. Chen, J. K. C. Toh, Y. L. Ang, J.-E. Jee, J. Lim, S. S. Lee and S.-G. Lee, *Chem. Sci.*, **2015**, 6, 450–456.

2 W. L. DeLano, *Schrödinger LLC*, **2002**, http://www.pymol.org.

3 M. J. Frisch, G. W. Trucks, H. B. Schlegel, G. E. Scuseria, M. A. Robb, J. R. Cheeseman, G. Scalmani, V. Barone, B. Mennucci, G. A. Petersson et al., *Gaussian 09, Revis. B.01, Gaussian, Inc., Wallingford CT*, **2009**.

4 A. Jakalian, D. B. Jack and C. I. Bayly, *J. Comput. Chem.*, **2002**, 23, 1623–1641.

5 D. A. Case, T. E. Cheatham, T. Darden, H. Gohlke, R. Luo, K. M. Merz, A. Onufriev, C. Simmerling, B. Wang and R. J. Woods, *J. Comput. Chem.*, **2005**, 26, 1668–1688.

6 J. A. Maier, C. Martinez, K. Kasavajhala, L. Wickstrom, K. E. Hauser and C. Simmerling, *J. Chem. Theory Comput.*, **2015**, 11, 3696–3713.

7 J. Wang, R. M. Wolf, J. W. Caldwell, P. A. Kollman and D. A. Case, *J. Comput. Chem.*, **2004**, 25, 1157–1174.

8 W. L. Jorgensen, J. Chandrasekhar and J. D. Madura, *J. Chem. Phys.*, **1983**, 79, 926–935.

9 I. Joung and T. Cheatham, *J. Phys. Chem. B*, **2008**, 112, 9020–9041.

10 T. Darden, D. York and L. Pedersen, *J. Chem. Phys.*, **1993**, 98, 10089–10092.

11 H. J. C. Berendsen, J. P. M. Postma, W. F. van Gunsteren, A. DiNola and J. R. Haak, *J. Chem. Phys.*

12 T. Soddemann, B. Dünweg and K. Kremer, *Phys. Rev. E*, **2003**, 68, 46702.

13 R. Salomon-Ferrer, A. W. G??tz, D. Poole, S. Le Grand and R. C. Walker, *J. Chem. Theory Comput.*, **2013**, 9, 3878–3888.

14 B. K. Ho, *Protein Sci.*, **2005**, 14, 1011–1018.

15 Y. K. Kang, J. S. Jhon and H. S. Park, *J. Phys. Chem. B*, **2006**, 110, 17645–17655.

16 W. S. Somers, J. Tang, G. D. Shaw and R. T. Camphausen, *Cell*, **2000**, 103, 467–479.

17 S. A. Samsonov, J. P. Gehrcke and M. T. Pisabarro, *J. Chem. Inf. Model.*, **2014**, 54, 582–592.

18 M. J. Abraham, T. Murtola, R. Schulz, S. Páll, J. C. Smith, B. Hess and E. Lindah, *SoftwareX*, **2015**, 1–2, 19–25.

19 A. W. Götz, M. J. Williamson, D. Xu, D. Poole, S. Le Grand and R. C. Walker, *J. Chem. Theory Comput.*, **2012**, 8, 1542–1555.

20 A. Onufriev, D. Bashford and D. A. Case, *Proteins Struct. Funct. Genet.*, **2004**, 55,

383–394.

21 S. Genheden and U. Ryde, *Expert Opin. Drug Discov.*, **2015**, 10, 449–461.

22 L. Borsig, R. Wong, J. Feramisco, D. R. Nadeau, N. M. Varki and A. Varki, *Proc. Natl. Acad. Sci. U. S. A.*, **2001**, 98, 3352–3357.
